# Modulation of TSIL-Based Ciprofloxacin Structures with pH for the Selective Extraction of Rare Earth Metals, Uranium, Thorium, Titanium, and Zinc

**DOI:** 10.1021/acsomega.5c13377

**Published:** 2026-03-17

**Authors:** David Lledó, Guillermo Grindlay, H. Q. Nimal Gunaratne, Abel de Cózar, Ana Sirvent, José M. Sansano

**Affiliations:** † Medalchemy, S. L. Ancha de Castelar, 46-48, entlo. A. San Vicente del Raspeig, Alicante 03690, Spain; ‡ Department of Analytical Chemistry, Nutrition and Food Sciences, University of Alicante, PO Box 99, Alicante 03080, Spain; § The QUILL Research Centre, School of Chemistry and Chemical Engineering, 1596 The Queen’s University of Belfast, Stranmillis Road, Belfast, Northern Ireland BT9 5AG, U.K.; ∥ Departamento de Química Orgánica I/Kimika Organikoa I Saila, Facultad de Química/Kimika Fakultatea, Universidad del País Vasco/Euskal Herriko Unibertsitatea (UPV/EHU) and Donostia International Physics Center (DIPC) and Centro de Innovación en Química Avanzada (ORFEO−CINQA), P. K, 1072, San Sebastián-Donostia 20018, Spain; ⊥ Ikerbasque, Basque Foundation for Science, Plaza Euskadi 5, Bilbao 48009 Spain; # Departamento de Química Orgánica, Centro de Innovación En Química Avanzada (ORFEO−CINQA) and Institute of Organic Synthesis, 16718 Universidad de Alicante. Ctra., Alicante-San Vicente s/n, Alicante 03080, Spain

## Abstract

We report a novel Task-Specific Ionic Liquid (TSIL) derived from ciprofloxacin in combination with CYPHOS·NTf_2_ that enables tunable selectivity toward the extraction of rare earth metal elements (REEs), thorium, uranium, titanium, and zinc. The highest metal-TSIL affinity was found at pH 6, showing the highest extraction coefficient values for thorium (446), uranium (404), Sc (307), and the remaining REEs, titanium, and zinc (238–71.5; 99; 98). Under highly acidic solutions, however, the TSIL only showed affinity for thorium but not for the remaining species. The metal-cation complexation has been studied by means of NMR spectroscopy and in silico DFT-based optimization of the complexes. The recyclability of the extractive system (up to seven extraction cycles) has also been proved.

## Graphical abstract

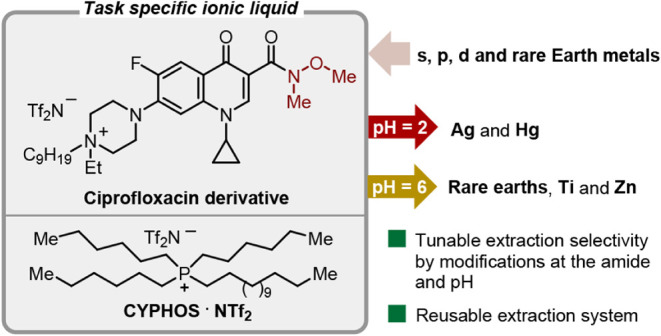

## Introduction

The recovery of rare-earth elements (REEs) from diverse sources has recently garnered significant attention. These 17 elements—comprising the 15 lanthanides along with scandium and yttrium—are relatively abundant and occur in numerous viable deposits globally.^1^ However, their availability is somewhat limited due to their extremely low concentration levels in many ores and the difficulty in their separation from each other. Rare-earth elements exert a profound influence on modern life; they are essential components in various technologies, ranging from the batteries of electric and hybrid vehicle motors to the high-performance magnets in automotive sound systems, permanent magnets, electrical sensors, catalysts, and phosphors in optical displays, media and communication devices (cell phones, televisions, computers, etc.), and instrumental analytical techniques. Their use is critical because their efficiency cannot be reproduced by other more abundant metals and, in fact, they are often referred to as “The Vitamins of Modern Industry”.^1^

On the other hand, uranium and thorium are involved in nuclear energy and also, to a lesser extent, in medicinal science. Thorium is typically more dispersed within host rock than uranium, necessitating more extensive mining, milling, and on-site processing at the point of recovery.^2^ Thorium is significantly more abundant in nature than uranium^2^ and is considered a “safer” alternative to uranium and plutonium. Consequently, it is frequently incorporated into reactor designs, particularly within accelerator-driven systems (ADS).^3^ Thorium is classified as a fertile material;^4^ although it is not fissile itself, it serves as a precursor to the fissile uranium ^233^U.

Methods used to separate and obtain f-block elements from the rest of the metals in the s, d, and p series are numerous^5^ and most of them are based on liquid–liquid extraction procedures. Other viable alternatives include the recycling mode of the hollow fiber renewal liquid membrane (HFRLM) process,^6^ the use of liquid membranes impregnated with *N,N,N′N′*-tetraoctyl-3-oxapentanediamide (TODGA) as an extractant,^7^ and high-performance liquid chromatography (HPLC).^8,9^

Within liquid–liquid extractions, there are processes that use a phosphate buffer,^10^ another that consists of a novel hydrophobic deep eutectic solvent-based dispersive liquid–liquid microextraction (DLLME),^11^ liquid–liquid extraction using fatty acids,^12^ a separation process based on the use of 2-[4-(2,4,4-trimethylpentan-2-yl)­phenoxy]­acetic acid (POAA),^13^ the use of a high-efficiency chelating agent by functionalizing graphene oxide with phenanthroline diamide (GO-PDA),^14^ using a binary extractor composed of 1,5-bis­[*o*-(dioxyphosphoryl)-*p*-ethylphenoxy]-3-oxapentane and an amine such as trioctylamine,^15^ *N*-*n*-heptylaniline,^16^ a hexaacetate-calix(6)­arene^17^ or a bidentate ligand, 2,3-dihydroxynaphthalene.^18^

The use of ionic liquids (ILs) has been fundamental in many examples of selective chemical separation, which has also been implemented in this area.^19^ Consequently, the application of ionic liquids (ILs) in conjunction with extractive ligands such as amines,^20^ 1,3-diketones (separation with SC–CO_2_),^21^ *N*,*N*-dialkylamides (separation with SC–CO_2_),^22^ diglycolamides,^23^ phosphine oxides (separation with oxalic acid),^24–27^ using phosphane-derived ligands (stripping with acidic solutions),^28^ methylimidazoles [excellent selectivities for uranium­(VI) and Ln­(III)],^29^ with trioctylphosphine oxide,^30^ with an aqueous solution of *N*,*N′*-dimethyl-*N*,*N′*-dioctyl-4-oxaheptanediamide (DMDOHA),^31^ with carbamoylmethylphosphine oxides (CMPO),^32,33^ with polymeric CMPO,^34^ *N*,*N*,*N′*,*N′*-tetraoctyldiglycolamide (TODGA) and other derivatives,^35,36^ tetradentate amides,^37^ and *N*,*N*-di­(2-ethylhexyl)-diglycolamic acid^38^ is extensively documented in the literature. Only ILs such as trioctylmethylammonium nitrate [A336]­[NO_3_][Bibr ref39] have been used alone.

Furthermore, room-temperature ionic liquids (RTILs) are increasingly viewed as viable alternatives to conventional molecular diluents for the extraction of metal ions from aqueous phases. Due to their superior radiolytic stability and enhanced extraction efficiencies, RTILs have been extensively investigated for the separation of both actinides and lanthanides.^40^ The extraction of actinide ions using room-temperature ionic liquid solvent systems—incorporating either diglycolamide (DGA) or functionalized DGA derivatives—has been extensively documented in the recent literature.^41^ Particularly assessed is the extraction of lanthanides­(III) and actinides­(III,IV) by task-specific ionic liquids.^42–47^

However, the use of so-called task-specific ionic liquids (TSILs) in combination with conventional ionic liquids has not been as frequent. A phosphoramidate-based TSIL has been used for the selective extraction of uranium.^48^ Another TSIL formed by an ionic liquid functionalized with 1-[3­[[(diphenylphosphinyl)­acetyl]­amino]­propyl]-3-tetradecyl-1*H*-3-imidazolium hexafluorophosphate was implemented for the general extraction of Th­(IV), U­(VI), and Ln­(III)^49^ and several similar TSILs were developed for the extraction of actinides.^50–54^

Processes involving TSILs, in combination with conventional ILs, have demonstrated synergistic effects, significantly improving the extraction efficiency, selectivity, and phase separation. To complete this extraction cycle, a highly efficient stripping stage is subsequently required.^55,56^

Currently, one of the most promising frontiers in f-element research involving ILs is their remarkable solvating capability toward actinides, lanthanides, and various fission products—all of which are critical constituents of nuclear waste and processing streams.^57^

Concerning REEs and radioactive elements, a very important application can match this strategy. The fertilizer industry also produces waste residues of several substances and elements. Specifically, the production of phosphate fertilizers, essential for global agriculture, is unfortunately coupled with the massive generation of a problematic byproduct known as phosphogypsum.^58^ Toxic heavy metals such as cadmium, arsenic, lead, and chromium, radioactive elements such as radium, thorium, and uranium, and REEs are also present in several proportions. Separating the toxic and radioactive components will cause: a) mitigation of environmental and health risks; b) the valorization of a waste product because a purified phosphogypsum product can be extensively used in the construction industry and agriculture; and c) resource recovery because the impurities themselves can be valuable.

Continuing with our studies based on the preparation of specific TSIL systems for selective metal extraction, particularly of thorium versus uranium,^59,60^ we propose that the design and computational simulation of a TSIL, tailored to the preferential coordination environment of a specific rare-earth element, is paramount. Such an approach would enable the precise modulation of separation selectivity and facilitate the implementation of a streamlined, environmentally benign protocol for TSIL/IL system recovery.^61^ Herein, we report the synthesis of a TSIL system derived from the antibiotic ciprofloxacin. We evaluate its performance in the selective extraction of REEs, uranium, thorium, zinc, and titanium, providing a comparative analysis against established TSIL systems.

## Results and Discussion

### Selected TSILs

Compounds featuring a 1,3-dicarbonyl moiety are well established as exceptional ligands, owing to their ability to stabilize metal complexes through effective chelation,^62^ specially, in the high coordination range of rare earth metals.^63^ Previous findings demonstrated that ionic liquids, functionalized with a 1,2-diamide motif, exhibited a strong coordinating effect, resulting in the quantitative extraction of the entire lanthanide series without significant selectivity.^38^ Our previous experience with the base structure of ciprofloxacin (**1**) in the selective extraction of thorium,^60^ and computational refined calculations, moved us to modify the electronic density of the 1,3-dicarbonyl fragment of the commercially available starting molecule **1**. *N*-alkylation of ciprofloxacin (**1**) was performed using ethyl bromide in an acetonitrile/water solvent system, with potassium iodide serving as a catalyst (Scheme 1). The resulting *N*-ethyl ciprofloxacin (**2**) was isolated in 92% yield. Subsequently, this compound underwent an amidation reaction with *N,O*-dimethylhydroxylamine. This reaction was facilitated by the in situ generation of the corresponding acid chloride, which was achieved using thionyl chloride in methylene chloride as the solvent at 0 °C for 30 min. The resulting Weinreb-type amide (**3**) was obtained in 95% yield.

**Scheme 1:**
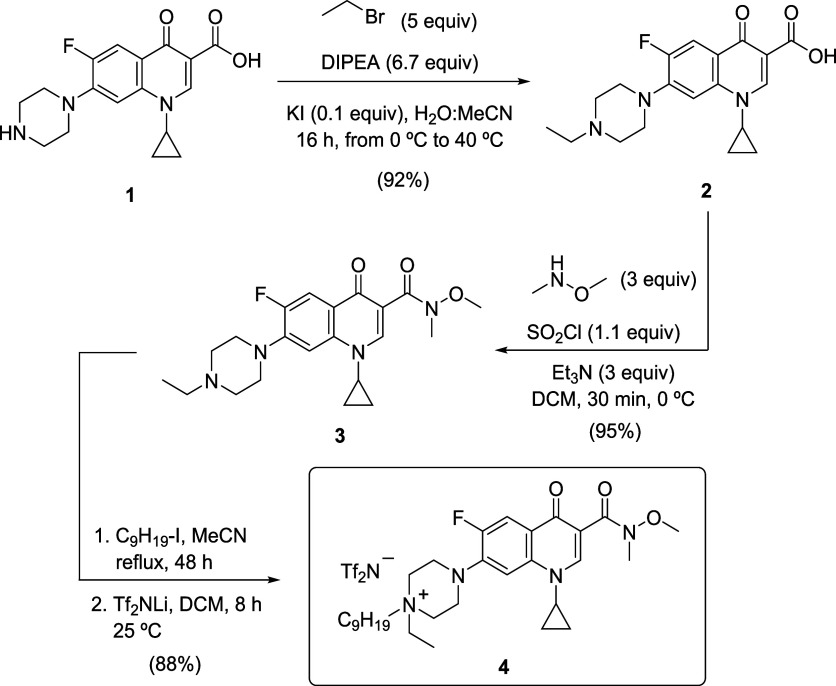
Synthesis of the TSIL **4**

The synthesis was concluded by the quaternization of the amine with nonyl iodide, followed by an anion exchange using lithium bis­(trifluoromethylsulfonyl)­imide in dichloromethane at room temperature for 8 h. These steps provided the final TSIL (**4**) with an 88% yield (76% cumulative yield from ciprofloxacin **1**) as shown in Scheme 1. The efficacy of this system is significant; the hydrophobic nature of the bis­(trifluoromethylsulfonyl)­imide anion (NTf_2_), combined with the long aliphatic chain on the ammonium cation, markedly improved metal partitioning into the organic phase and facilitated efficient solvent recovery.

Together with TSIL **4**, other TSILs previously reported by our group, such as **6** ^64^ and **7**,^60^ and the commercially available IL **5**, will be assessed in this work (Figure 1). In all these cases, CYPHOS·NTf_2_ IL (**5**) was used as the solvent of TSILs **4**, **6**, and **7**.

**Figure 1:**
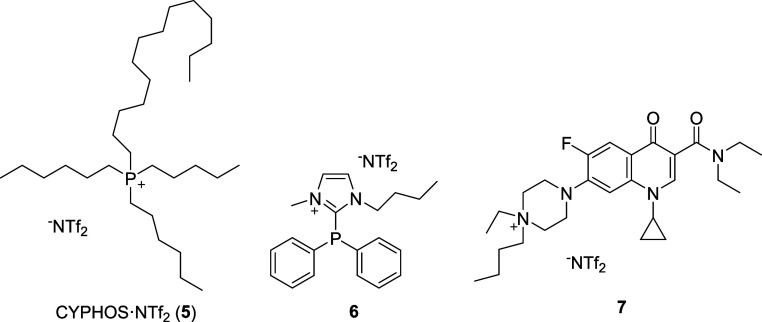
Other IL employed in this work.

### Microextraction Tests

A preliminary evaluation of the general extractive properties of TSILs **4**, **6**, and **7** was conducted using lanthanum as a metallic reference. This assessment was based on the previously observed general affinity of ciprofloxacin-derived TSILs toward various REEs, thorium, and uranium.^60^ The extractive capabilities of ionic liquid **5** itself and 0.005 M solutions of TSILs **4**, **6**, and **7** in **5** were compared (Figure 2).

**Figure 2:**
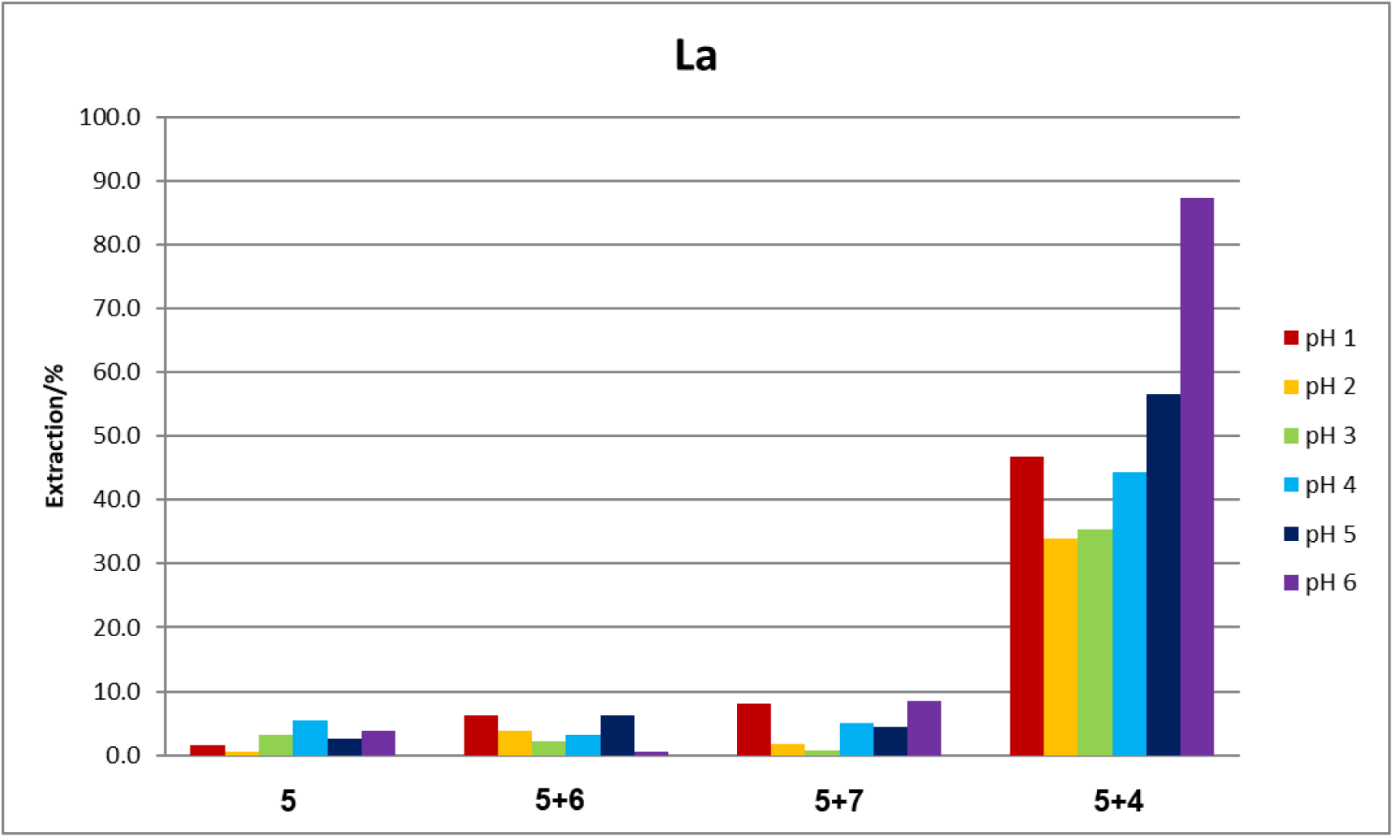
Comparative extractions of lanthanum by IL systems tested in this work.

When we tested a multimetallic sample containing all the REE, uranium, and thorium, lanthanum (III) nitrate was noticeably extracted by the mixture **5** + **4** rather than the other TSIL combinations from buffered aqueous solutions specially at pH = 6 (Table 1). Similar findings were found for other REEs as well as for thorium and uranium (Supporting Information). It is noticeable the importance of the IL (**5**) used as a solvent is in these methodologies, particularly for thorium. While BMIM·NTf_2_+ **7** selectively extracted this element at pH = 1,^60^ the combination **5** + **4** is able to extract completely thorium in all pH range of study (see Supporting Information).

**Table 1:** Extraction Coefficients K Determined in the Microextractive Experiment at 25 °C

Metal	K, pH 2 with **5**	K, pH 2 with **5** + **4**	K, pH 6 with **5**	K, pH 6 with **5** + **4**
Sc	1.6	25.9	<1	**307.0**
Y	<1	<1	<1	**71.5**
La	<1	12	<1	**88.4**
Ce	<1	<1	<1	**101.3**
Pr	<1	1.1	<1	**95.4**
Nd	<1	1.0	<1	**94.5**
Sm	<1	1.4	<1	**96.7**
Eu	<1	1.3	<1	**94.1**
Gd	<1	<1	<1	**101.3**
Tb	<1	<1	<1	**98.7**
Dy	<1	<1	<1	**98.7**
Ho	<1	<1	<1	**102.0**
Er	<1	1.0	<1	**99.0**
Tm	<1	2.0	<1	**193.4**
Yb	<1	4.4	<1	**235.2**
Lu	<1	12.3	<1	**238.2**
Th	3.3	**103.4**	15.9	**446.2**
U	2.1	12.6	4.7	**404.0**
Li	<1	<1	<1	<1
B	<1	<1	<1	<1
Al	<1	<1	<1	<1
Ti	2.0	1.6	3.6	**99.2**
V	<1	<1	<1	<1
Cr	<1	<1	1.2	1.7
Mn	<1	<1	<1	<1
Fe	<1	<1	4.8	5.6
Co	<1	<1	<1	<1
Ni	<1	<1	<1	<1
Cu	<1	<1	4.0	11.2
Zn	<1	<1	**96.2**	**98.4**
Ga	<1	<1	2.3	4.0
As	<1	<1	<1	<1
Se	<1	<1	<1	<1
Zr	19.7	32.3	1.6	1.7
Mo	1.1	<1	<1	<1
Ru	<1	<1	<1	<1
Pd	<1	7.3	<1	1.8
Ag	24.3	**49.4**	19.7	32.1
Cd	<1	<1	<1	<1
In	8.2	8.2	2.6	4
Sn	<1	<1	2.5	3.9
Sb	<1	<1	<1	<1
Te	<1	<1	1.2	2.0
Pt	<1	<1	<1	<1
Au	<1	<1	<1	<1
Tl	<1	<1	<1	<1
Hg	24.2	**99.0**	16.0	24.0
Pb	<1	<1	1.9	3.0
Bi	13.3	15.7	1.5	2.3

For all the aforementioned elements, the distribution ratio K, defined as the ratio of the metal concentration in the organic phase to that in the aqueous phase at equilibrium (eq 1), was determined under various experimental conditions:

1 $$K=\mathrm{Final}{[M]}_{\mathrm{org}}/\mathrm{Final}{[M]}_{\mathrm{aq}}$$

These extraction coefficients under both optimum (pH= 6) and nonoptimum extraction conditions (pH= 2) using the **5** + **4** couple are shown in Table 1. As expected from previous experiments, TSIL extraction coefficients vary significantly with pH (Table 1) and the solvent contribution to the extraction, **5**, was negligible. Under highly acidic conditions (pH = 2), extraction coefficients for all the REEs were not particularly high except for Sc (25.9), La (12), and Lu (12). However, extraction coefficient values significantly increased at a pH of 6 (>71.5). The highest values were obtained again for Sc (307) and Lu (238.2) but other species showed similar values such as Tm (193.4) and Yb (235.2). For the remaining REEs, coefficient values were similar (approximately 90). In agreement with previous observations, both thorium and uranium showed the highest extraction coefficients at pH 6, being significantly higher than those previously shown by the REEs (>400). Interestingly, the extraction coefficient for thorium at pH 2 was important (103.4) and higher than the one shown by uranium. So, a priori, both species could be selectively separated by the appropriate selection of pH with the **5** + **4** couple.

To investigate TSIL **4** selectivity in depth, the extraction coefficients for several transition metals and metalloids were also determined under equivalent conditions to those previously employed for REEs, thorium, and uranium. Thus, a 0.005 M solution of TSIL **4** in **5** (100 μL) was performed (at 25 °C) from a multimetal sample (5 mL) (Li, B, Al, Ti, V, Cr, Mn, Fe, Co, Ni, Cu, Zn, Ga, As, Se, Zr, Mo, Ru, Pd, Ag, Cd, In, Sn, Sb, Te, Pt, Au, Hg, Tl, Pb, and Bi). It was observed that Ti and Zn behaved similarly to REEs and could be selectively extracted at pH 6 with the **5** + **4** couple. Under highly acidic conditions (pH 2), however, TSIL **4** showed some affinity against some species such as Hg, Ag, and Zr. Nevertheless, these elements were also partially extracted by the CYPHOS·NTf_2_ (**5**) itself. Similarly, it was also observed that Zn was efficiently extracted by the CYPHOS·NTf_2_ (extraction coefficient >96) but not the TSIL **4**. A possible mechanism that justified this exclusive extraction was a simple anion exchange process, which was favored by the ionic character of the ILs, such as it was reported in the literature.^65–68^ From these results, it is evident that, by the appropriate selection of given TSIL and IL solvent media, metal extraction capabilities can be tunable.

The pH remains a critical parameter for the precise fine-tuning of rare-earth element (REE), thorium, and uranium extraction. Although direct background references for comparing these specific results are not available in the literature, numerous studies have addressed the influence of pH on the extraction process. For instance, the effective extraction of Y, La, Nd, and Sm was achieved exclusively at pH 5 using a Cyphos IL 104-supported membrane.^69^ Several authors suggested that the extraction proceeds via an anion-exchange mechanism.^69^ Alternatively, the CYPHOS IL 104-supported membrane may undergo acidification to form the HX·IL, in which both the cation and anion are directly involved in REE extraction through a coordination mechanism. These mechanisms may explain our results in the absence of the TSIL (**4**) component.^19,69^ However, the 1,3-dicarbonyl arrangement of **4** acts as a Pearson hard base in nonacidic media, favoring the complexation of hard REE, uranium, and thorium cations. Presumably, increasing the proton concentration (H^+^ can be a competitor of M^+x^) leads to the protonation (partial blocking) of this dicarbonyl group in the ciprofloxacin moiety, thereby hampering its coordination with the REEs. It can be a plausible explanation of the exclusive extraction of Th at pH = 2 here and at pH = 1 using a diethyl amide of the ciprofloxacin.^60^ In addition, the results of this work under pH = 6 (using Weinreb amide **4**) are not so selective due to the absence of protons in the system. This behavioral difference does not depend exclusively on proton concentration; rather, the electron density of the 1,3-dicarbonyl system also provides a significant contribution.

### Selective Extraction of Titanium and Zinc (Hg)

While our main objective was the study of REEs, thorium, and uranium extraction, the unexpected behavior of titanium and zinc warrants an initial discussion due to their significant interest. Titanium, whose importance stems from its unique ability to solve modern problems such as making things lighter to save energy and resisting corrosion in the harshest environments on Earth, has been selectively extracted using ILs,^70–72^ or IL + DES combination.^73^ However, no publications have been edited on the basis of the extractive concept using an IL + TSIL system.

Zinc—apart from galvanization processes—has evolved into a strategic material essential for global food security and the green energy transition. It is currently the backbone of high-yield agriculture through biofortified fertilizers that combat soil deficiency, while simultaneously emerging as a safer, fireproof alternative to lithium for massive grid-scale energy storage. For the selective zinc metal extraction (together with other metals) the employment of IL is very frequent.^74–78^ Employment of IL + TSILs^79–82^ afforded very important selectivities with notable recovery parameters. These TSIL structures were focused on the green design of hydrophilic, low cost, and simple organic molecules.

Finally, the high extraction potential of **5** + **4** observed at pH = 2 for mercury can be an extraordinary result to implement this methodology as a sensor for detecting traces of this metal in solid food.^83^

### ^1^H NMR Analyses of Scandium­(III), Ytterbium­(III), Thorium­(IV), Lanthanum­(III), and Lutetium­(III) Complexes

The complexation of metal cations with TSIL **4** was investigated by using ^1^H NMR spectroscopy. To represent the wide range of extracted metals, we selected scandium­(II), ytterbium­(III), thorium­(IV), lanthanum­(III), and lutetium­(III) nitrates as representative examples. The maximum inner-sphere coordination number of the TSIL **4** ligand per cation was determined by monitoring the coordination-induced chemical shifts of the signals at 8.01 ppm (s) and 7.80 ppm (d). Notably, the doublet at 7.50 ppm remained largely unaffected upon the addition of various metallic cations, suggesting its minimal involvement in the metal-binding environment (Figure 3). Significant signal shifts (ppm) were observed up to a 3:1 **4**:Yb^3+^ ratio, indicating the formation of the most stable species in deuterated DMSO.

**Figure 3:**
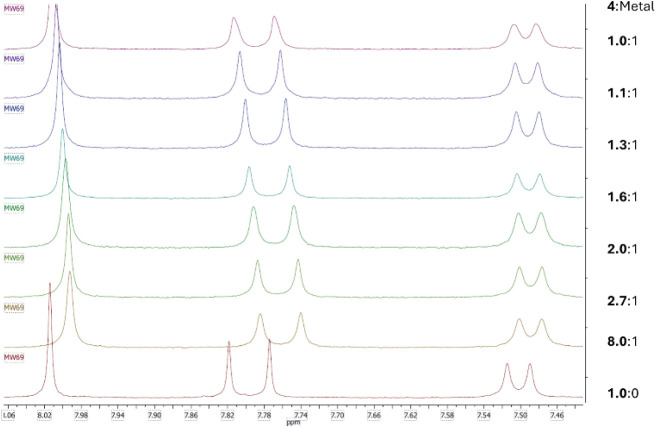
Stack plot of the titration results of ytterbium­(III) nitrate with **4** monitored by ^1^H NMR spectroscopy.

The complexation process was further visualized by plotting the chemical shift difference Δδ (difference in the chemical shift = δ_4·M_ – δ_4_) versus the concentration of the **4**:M^+x^ complex (in mmol·mL^–1^) as shown in Figure 4. This quantitative analysis revealed a clear preference for a 3:1 ligand-to-cation ratio for all five nitrates tested (scandium, ytterbium, thorium, lanthanum, and lutetium). This coordination pattern remains consistent regardless of the number of coordinating dimethyl sulfoxide (DMSO-d_6_) molecules solvating the metal cations. These findings are also in agreement with previously reported data for an analogous ciprofloxacin-derived TSIL.^60^ Regardless of the specific coordination numbers of the individual metal ions, three TSIL **4** ligands consistently coordinate to the metallic outer sphere.

**Figure 4:**
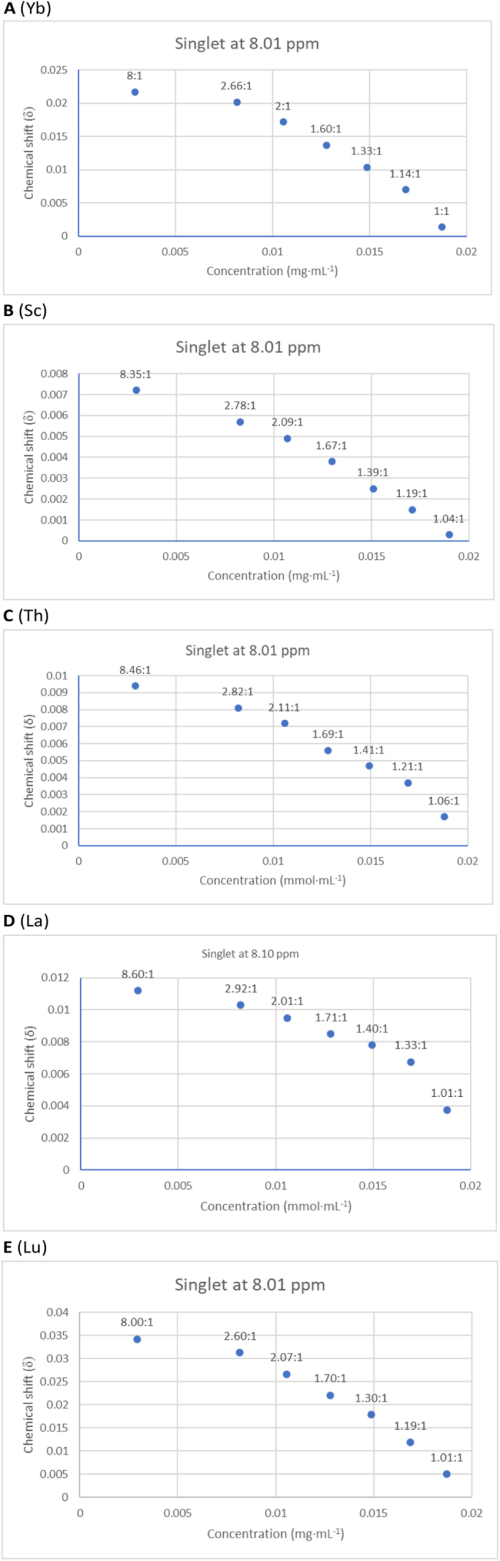
Plot of the variation of the chemical shift (in ppm) of the singlet observed at 8.01 ppm versus the concentration of the TSIL **4** during the titration of: (A) ytterbium­(III) nitrate; (B) scandium­(III) nitrate; (C) thorium­(IV) nitrate; (D) lanthanum­(III) nitrate; (E) lutetium­(III) monitored by ^1^H NMR spectroscopy.

### DFT-Based Computational Optimization of the Complexes

Due to the challenges in obtaining suitable single-crystal X-ray diffraction data, we turned to computational modeling to evaluate the observed coordination preferences. The coordination motifs applied in these simulations are consistent with the titration results derived from the ^1^H NMR experiments discussed previously. Thorium­(III)^84^ and uranium­(IV) were deeply analyzed in our previous contribution.^60^ Here, thorium exhibited an extraordinary preference for this ciprofloxacin-derived TSIL **4** (see Table 1, K = 446.2), but the rare-earth elements also have a high affinity for the TSIL **4**, so the different interaction of the lanthanum­(III) and lutetium­(III) nitrates with the ligand **4** will be analyzed and compared as representative examples in this work following an analogous conceptual scheme.^60^

It is well established that the coordination chemistry of lanthanum is notoriously complex, characterized by structural plasticity and a broad range of potential coordination numbers, which significantly complicates the definitive characterization of these species.^85^ The same situation was found in our previous assessment of the preference of thorium­(IV) toward an octahedral arrangement considering ciprofloxacin-derived ligands.^60^ Considering these facts and the titration of the cationic species using TSIL **4** (Figure 4D and E), the coordination number of the assessed compounds was initially set to 10 and the relative energies, corresponding to the replacement of molecules of water by units of TSIL (ligand) **4** in an aqueous environment, were analyzed. Our study is based on the hypothesis that the extraction coefficients of La and Lu are related with the water-ligand (**4**) exchange equilibrium (Scheme 2).^58^ In view of the experimental results (*vide supra*), only metal-dinitro polyaquo species, capable of generating compounds with a 1:3 metal:**4** ratio, were considered.

**Scheme 2:**
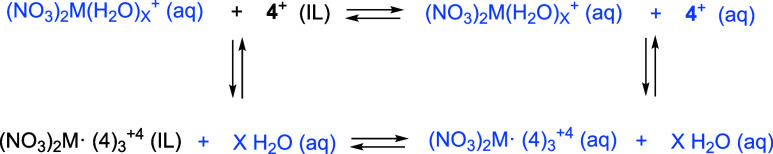
Simplified Model Used for DFT Calculations of La/Lu· **4**_3_ Formation

Due to the complexity of calculations of rare-earth elements and the number of metals involved in this study, we have selected lanthanum and lutetium to justify the difference between their extraction constants (K) depicted in Table 1 and, also, the good affinity of both metals to the TSIL. In the initial exploration, the polyaquo species show a difference between La and Lu compounds. While La presents a coordination number of 10, where the coordination sphere of the dinitro compounds is fulfilled by six molecules of water, Lu prefers a coordination number of 9, where only five molecules of water complete the inner coordination sphere of the metal (Figure 5).^86^

**Figure 5:**
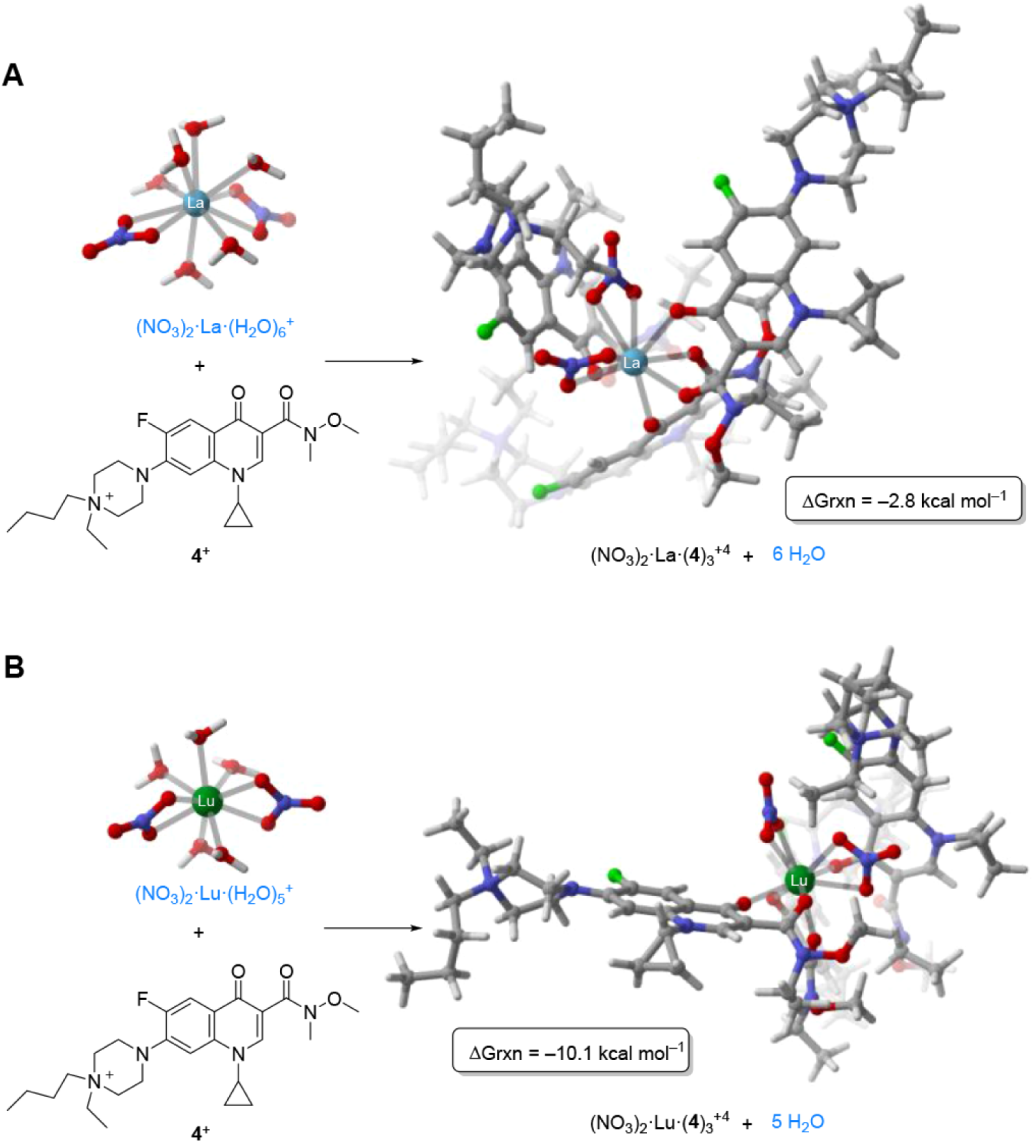
Reaction Gibbs energies associated with the water—**4** exchanges of (A) La­(III) and (B) Lu­(III) nitrate complexes computed at the DFT level.

The computed reaction Gibbs energies indicate that for La the water ligand exchange is thermodynamically slightly favored, as reflected by the value of −2.8 kcal mol^–1^, in good agreement with the moderate extraction coefficients obtained experimentally. On the other hand, the energy value obtained for Lu (−10.1 kcal mol^–1^) would correspond to a higher extraction coefficient. It is worth to mention that the obtained reaction Gibbs energies are considerably lower than the ones obtained for Th complexes using related diethylamido ligands (−37.8 kcal mol^–1^ for the octahedral Th complexes).^60^ We hypothesize that this difference can be related to the existence of the *N-*methoxy amide moiety in TSIL **4** (not present in the previous study), which may affect the electronic density of the amide oxygen atom that coordinates to the metal center.

### Kinetic Analysis and Loading Tests

The kinetics of the affinity of TSIL **4** toward scandium­(III), lutetium­(III), ytterbium­(III), and thorium­(IV) cations were evaluated at pH 6 using Inductively Coupled Plasma (ICP) as the detection technique. In these experiments, 1 mL of an aqueous 0.005 M solution of each nitrate salt (0.02 mmol of total cations) was extracted with 1 mL of a 0.1 M solution of **4** in CYPHOS-NTf_2_ IL (**5**), which provided 0.1 mmol of **4** (Figure 6). The relative affinity observed in Figure 6 is consistent with the results presented in Table 1. Specifically, thorium and scandium demonstrated higher K values, whereas the extraction kinetics for ytterbium and lutetium were very similar to each other.

**Figure 6:**
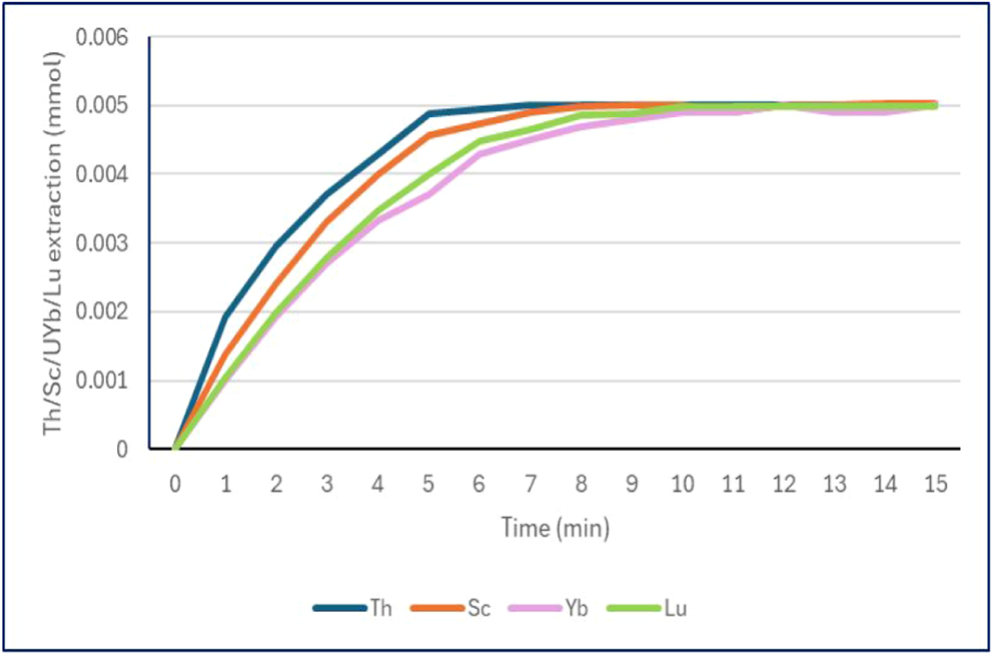
Kinetics of the extraction by **4** in **5** (0.1 mmol of **4**/mL of **5**) of a) thorium­(IV) (blue plot); b) scandium­(III) (orange plot); c) ytterbium­(III) (pink plot); and d) lutetium­(III) (green plot).

### Optimal Loading of Extractive Solution and Recycling Studies

The optimal loading and cycling stabilities of the extractive mixture were determined to ensure efficient recovery. This was established based on the methodology developed for a previously published ciprofloxacin-derived TSIL system.^60^ To perform the extraction, an aqueous buffered solution of the metal nitrate (solution A, pH 6) was mixed with the ionic liquid phase (solution B) and stirred for 12 min. Following this, the two phases were separated in situ: the metal-free aqueous phase (A) and the ionic liquid phase (B) containing the extracted rare earth metal, thorium, or uranyl cation (Figure 7).

**Figure 7:**
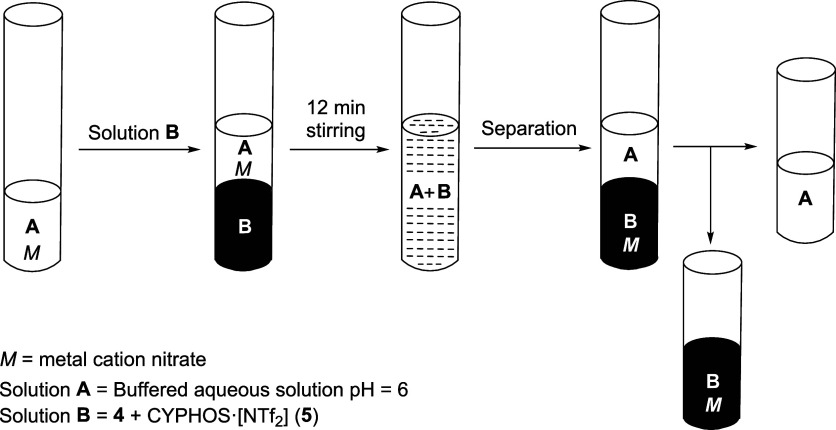
General sequence of the selective extraction of metal cations of rare-earth elements, uranium, or thorium.

The stripping process, designed to recover the metal-free ionic liquid (IL) phase (B), was conducted as illustrated in Figure 8. Nitric acid at pH 0.5 (solution C) was introduced to solution B, and the mixture was stirred for 6 min. ICP analysis confirmed a 98% extraction yield, demonstrating that the regenerated IL phase could be successfully recycled for subsequent extraction cycles.

**Figure 8:**
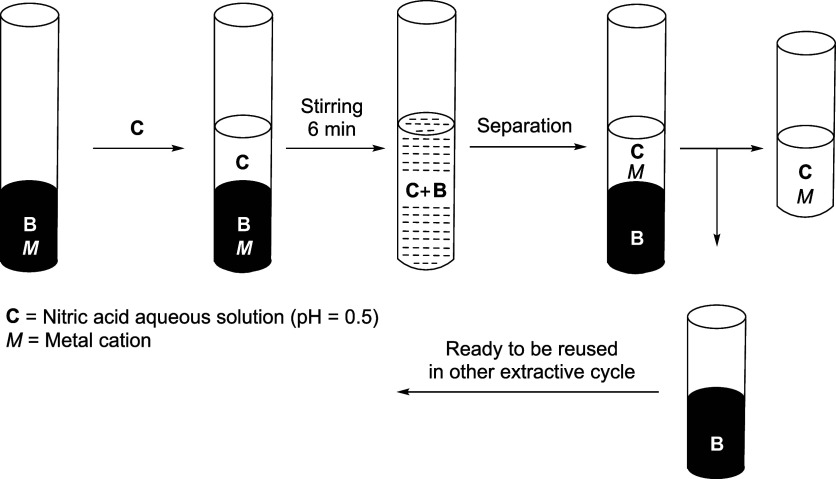
General procedure for the selective stripping of the cation involved in the extraction.

The optimal concentration for the recycling studies was determined to be 0.3 M, consistent with previous reports on the selective extraction of thorium over uranium.^60^ Using these parameters, recovery tests for Yb, Lu, Sc, and Th (Figure 9) demonstrated excellent chemical recovery yields for up to the seventh cycle of the process with no notable decrement in performance.

**Figure 9:**
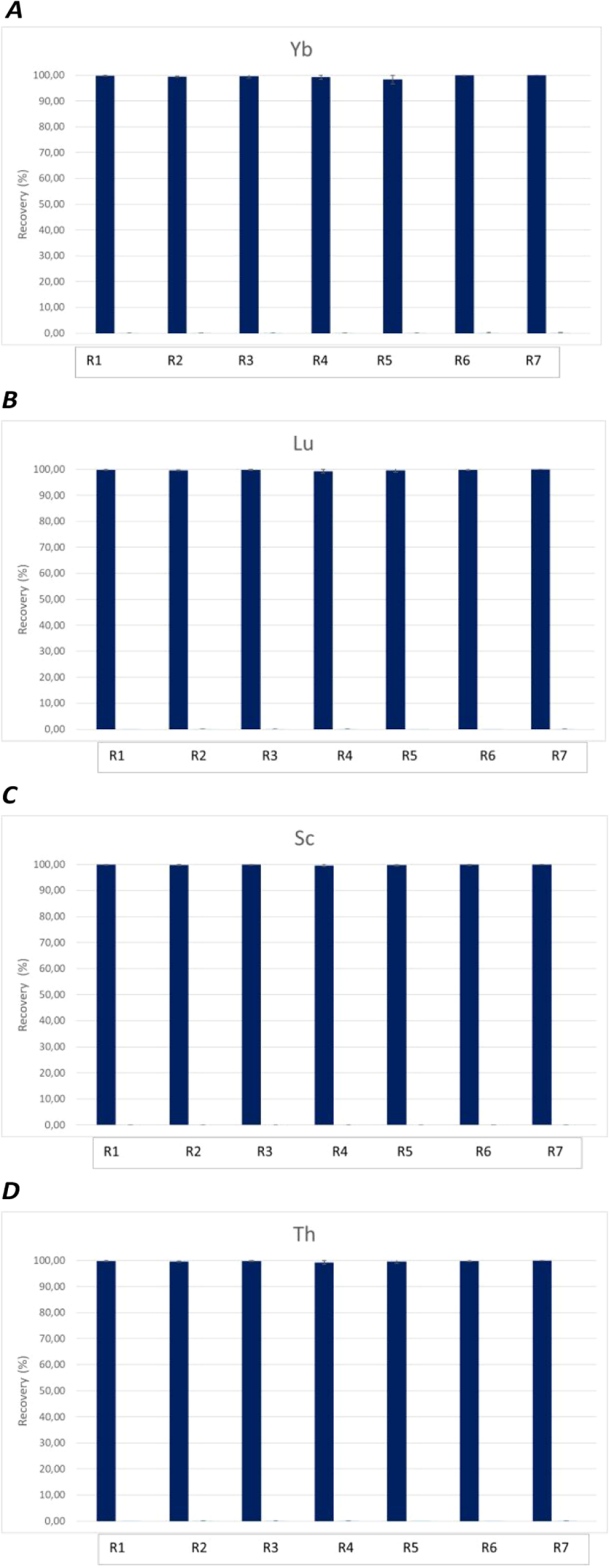
Recovery tests of **4** + **5** for A) Ytterbium; B) Lutetium; C) Scandium, and D) Thorium.

## Conclusions

In 2026, most of the metals extracted in this article became geopolitical and industrial cornerstone. Our research demonstrates that a solution of TSIL **4** in CYPHOS·NTf_2_ (**5**), at pH = 6, excels in the selective extraction of REEs, thorium, uranium, and titanium, exhibiting its strongest affinity for thorium. We have shown that by precisely tuning the substituent of the 1,3-dicarbonyl arrangement and the pH, the coordination strength to the metal can be modified, resulting in tunable selectivity (also justified by computational studies). These findings significantly broaden the scope of selective extraction compared to our group’s prior publication.^60^ Our findings indicate that each metal atom is directly complexed by three TSIL units. This structural arrangement and the high affinity observed experimentally were both corroborated by simplified aqueous models derived from DFT calculations. A stock solution of TSIL **4** in ionic liquid (IL) was found to be stable at a concentration of 0.3 M for extraction purposes. To ensure the system reached equilibrium, a stirring period of 12–13 min was required. Furthermore, the recyclability of the resultant IL mixture proved excellent for all metals investigated, underscoring the inherent robustness of the mixture and the hydrophobicity contributed by CYPHOS·NTf_2_ (**5**). The same **4** + **5** mixture can be effectively used for over seven extraction batches. This number is the highest reported in the literature until now in the examples of the extractions or REEs, Th, U, Ti, Zn, and also Hg. This advanced prototype is now positioned for diverse industrial applications, ranging from the selective recovery of critical metals in mining operations to the upcycling of electronic waste. Furthermore, it holds significant potential for the remediation of complex radioactive waste streams within the nuclear energy sector. In parallel studies performed at pH = 2 the **4** + **5** mixture extracted selectively thorium and showed some affinity toward other species such as mercury (which is applicable as an Hg sensor in food), Ag, and Zr. These conditions reproduced our results obtained previously attaining thorium extraction. A small difference in the electron density around the 1,3-dicarbonyl moiety (**7** versus **4**) is crucial to define the selective extraction. It is particularly noteworthy the ability of CYPHOS·NTf_2_ (**5**) itself to extract selectively zinc at pH = 6. The sum of an anionic interchange and a coordination of the phosphorus atom can be the origin of this result.

We propose that this selective extraction protocol could be effectively adapted for the valorization of phosphogypsum, which is a major byproduct of the phosphate fertilizer industry. This material is generated during phosphoric acid production via the sulfuric acid digestion of phosphate rock, and our method offers a pathway for recovering critical elements from these vast industrial streams. The high contents of radioactive elements, such as thorium and uranium, REEs, mercury, silver, etc., would be trapped, generating a very pure and nontoxic phosphogypsum ready to be used safely. This phosphogypsum is typically stored in large, dedicated gypsum stacks that pose risks to groundwater and air quality.

## Experimental Section

### General Remarks

All of the reagents and solvents were purchased from commercial suppliers and used without further purification. Low-resolution mass spectra (EI) were obtained using an Agilent GC/MS 5973N spectrometer at 70 eV; fragment ions are reported in *m*/*z* with relative intensities (%) in parentheses. NMR spectra were recorded on Bruker AV300 Oxford, Bruker 400 Neo Ascend, or Bruker AV400 spectrometers (^1^H at 300 or 400 MHz; ^13^C at 75 or 100 MHz; ^19^F at 282 or 376 MHz). Spectra were acquired in CDCl_3_ using TMS as an internal standard (0.00 ppm). Data are reported as follows: s (singlet), d (doublet), t (triplet), q (quartet), m (multiplet or unresolved), br s (broad signal), coupling constants (*J*) in Hz, and integration. ^13^C and ^19^F NMR spectra were recorded with ^1^H-decoupling and referenced to CDCl_3_ (77.16 ppm) and CF_3_CO_2_H (−76.6 ppm), respectively. Elemental analyses (EA) were performed using a TruSpec CN with an S module and a FlashSmart CHNS instrument (Thermo Scientific). ICP and microanalyses were obtained via PerkinElmer Optima 7300 DV and LECO Micro TruSpec instruments, respectively.

#### Synthesis of 1-cyclopropyl*-*7*-*(4-ethylpiperazin-1-yl)*-*6*-*fluoro-4-oxo-1,4-dihydroquinoline-3-carboxylic Acid (**2**)^60^

To a solution of ciprofloxacin hydrochloride (3.1 g, 8.0 mmol) and DIPEA (6.7 equiv, 9.3 mL, 6.9 g, 54 mmol) in MeCN:H_2_O (1:1, 30 mL/mmol, 240 mL), bromoethane (5.0 equiv, 3.0 mL, 4.4 g, 40 mmol) was added dropwise at 0 °C. Potassium iodide (10 mol %, 132 mg, 0.8 mmol) was then added at the same temperature. The mixture was stirred at 0 °C for 1 h and then it was heated to 40 °C and stirred at this temperature for 16 h. The solvent was removed under reduced pressure, and the residue was dissolved in DCM (40 mL), washed with a phosphate buffer solution at pH = 6.5 (30 mL) and dried with MgSO_4_. The solvent was removed under reduced pressure and pure compound **2** was obtained without further purification (2.6 g, 7.4 mmol, 92%) as a white solid; ^1^H NMR (300 MHz, CDCl_3_) δ 8.75 (s, 1H), 7.99 (d, *J* = 13.1 Hz, 1H), 7.36 (d, *J* = 7.1 Hz, 1H), 3.58–3.52 (m, 1H), 3.39–3.34 (m, 4H), 2.73–2.65 (m, 4H), 2.52 (q, *J* = 7.2 Hz, 2H), 1.42–1.32 (m, 2H), 1.22–1.18 (m, 2H), 1.15 (t, *J* = 7.2 Hz, 3H), *Proton from the carboxylic acid was not observed; ^13^C NMR (101 MHz, CDCl_3_) δ 177.2, 167.8, 153.8 (d, *J* = 252.5 Hz), 147.5, 146.1 (d, *J* = 10.1 Hz), 139.2, 119.8 (d, *J* = 8.1 Hz), 112.4 (d, *J* = 24.2 Hz), 108.2, 104.9, 52.5 (2C), 52.4, 49.9 (2C), 35.4, 12.0, 8.3 (2C); ^19^F NMR (282 MHz, CDCl_3_) δ −120.6 (s, 1F); IR (neat) ν_max_ 3420–3395, 1707–1675 cm^–1^; LRMS (EI) *m*/*z* 360 (M^+^+1, 16%), 359 (M^+^, 69%), 344 (24), 316 (21), 315 (100), 287 (13), 257 (13), 230 (12), 150 (10), 84 (24), 70 (12), 57 (32), 56 (11), 44 (27), 42 (19); Elemental analysis calcd for C_19_H_22_FN_3_O_3_: C, 63.50; H, 6.17; F, 5.29; N, 11.69; and O, 13.35. Found: C, 63.3; H, 6.5; N, 11.5.

#### Synthesis of 1-cyclopropyl-*N*-methoxy-*N*-methyl-7-(4-ethylpiperazin-1-yl)-6-fluoro-4-oxo-1,4-dihydroquinoline-3-carboxamide (**3**)

To a solution of **2** (546 mg, 1.50 mmol) and Et_3_N (3.00 equiv, 0.64 mL, 0.45 g, 4.50 mmol) in DCM (3 mL), *N,O-*dimethylhydroxylamine (3.00 equiv, 0.35 mL, 275 mg, 4.50 mmol) and thionyl chloride (1.10 equiv, 0.12 mL, 196 mg, 1.65 mmol) were added dropwise at 0 °C and the mixture was stirred at this temperature for 30 min. The solvent and volatile compounds were removed under reduced pressure. The residue was dissolved in CHCl_3_ (30 mL), washed with a saturated aqueous solution of sodium bicarbonate (3 × 15 mL), then washed again with a saturated aqueous solution of potassium carbonate (3 × 10 mL) and dried with MgSO_4_. The solvent was removed under reduced pressure and the residue was purified by recrystallization with Et_2_O to yield pure compound **3** (573 mg, 1.42 mmol, 95%) as a sticky yellowish solid–liquid; ^1^H NMR (400 MHz, CDCl_3_) δ 8.67 (s, 1H), 7.91 (d, *J* = 4.0 Hz, 1H), 7.30 (d, *J* = 7.1 Hz, 1H), 3.67 (s, 3H), 3.57–3.50 (m, 1H), 3.45–3.25 (m, 4H), 3.27 (s, 3H), 2.80–2.67 (m, 4H), 2.52–2.45 (q, *J* = 7.2 Hz, 2H), 1.40–1.20 (m, 2H), 1.28–1.23 (m, 2H), 1.14–1.09 (m, 3H); ^13^C NMR (101 MHz, CDCl_3_) δ 172.4, 165.4, 153.3 (d, *J* = 248.1 Hz), 144.5 (d, *J* = 11.3 Hz), 142.9, 138.6, 121.8 (d, *J* = 7.0 Hz), 119.4, 112.7 (d, *J* = 22.7 Hz), 104.8, 63.9, 61.3, 52.7, 52.4 (2C), 50.1 (2C), 34.3, 12.1, 8.2 (2C); ^19^F NMR (282 MHz, CDCl_3_) δ −123.6 (s, 1F); LRMS (EI) *m*/*z* 402 (M^+^, 11%), 342 (12), 302 (20), 303 (100); Elemental analysis calcd for C_21_H_27_FN_4_O_3_: C, 62.7; H, 6.8; F, 4.7; N, 13.9. Found: C, 62.6; H, 7.0; N, 13.9.

#### Synthesis of bis­(trifluoromethane)­sulfonimide 1-cyclopropyl-7-(4-ethyl-4-nonyl-4lamda4-piperazin-1-ylinium)-6-fluoro-*N*-methoxy-*N*-methyl-4-oxo-1,4-dihydroquinoline-3-carboxamide Salt (**4**)

A solution of **3** (207 mg, 0.50 mmol) and iodononane (2.00 equiv, 0.20 mL, 254 mg, 1.00 mmol) in MeCN (8 mL) was stirred at 80 °C for 48 h. The solvent was removed under reduced pressure. The residue was dissolved in CHCl_3_ (10 mL), an aqueous solution of lithium bistriflimide (1.30 equiv, 0.65 mmol, 0.5 M, 1.3 mL) was added, and the mixture was stirred at 25 °C for 15 h. Then, the aqueous phase was extracted with CHCl_3_ (15 mL) and the combined organic fractions were washed with H_2_O (2 × 10 mL) and dried with MgSO_4_. The solvent was removed under reduced pressure and the residue was purified by recrystallization with EtOAc to yield pure compound **4** (356 mg, 0.44 mmol, 88%) as a sticky solid–liquid; ^1^H NMR (400 MHz, CD_3_CN) δ 7.97 (s, 1H), 7.77 (d, *J* = 15.0 Hz, 1H), 7.43 (d, *J* = 7.5 Hz, 1H), 3.62 (s, 3H), 3.56 (s, 6H), 3.48 (q, *J* = 7.3 Hz, 4H), 3.38–3.32 (m, 2H), 3.21 (s, 3H), 1.74–1.68 (m, 2H), 1.41–1.15 (m, 18H), 1.09–1.05 (m, 2H), 0.92–0.87 (m, 3H); ^13^C NMR (101 MHz, CD_3_CN) δ 173.1, 166.7, 153.8 (d, *J* = 245.5 Hz), 142.9 (d, *J* = 11.0 Hz), 139.7, 125.7, 122.5 (d, *J* = 6.6 Hz), 120.3 (q, *J* = 259.0 Hz, 2C), 116.2, 112.6 (d, *J* = 21.9 Hz), 107.5, 61.6, 58.6, 54.8, 44.0 (2C), 35.2, 34.2 (2C), 32.5, 30.0, 29.8, 29.1, 27.0, 23.3, 21.9, 14.3 (2C), 8.6, 7.5 (2C); ^19^F NMR (282 MHz, CD_3_CN) δ −77.5 (s, 6F), −124.4 (s, 1F); LRMS (EI) *m*/*z* 529 (M^+^, 2%), 315 (100), 271 (33). Elemental analysis calcd for C_32_H_46_F_7_N_5_O_7_S_2_: C, 47.5; H, 5.7; F, 16.4; N, 8.7; S, 7.9. Found: C, 47.1; H, 5.5; N, 9.1; S, 7.8.

### General Procedure for the Multimetallic Analysis (Figure 2)

A microextractive multimetallic survey was conducted at 25 °C using an extraction system composed of a 0.005 M solution of TSIL **4** in **5** (100 mL). Two types of samples were evaluated: (a) a multimetal sample containing REEs, uranium, and thorium; and (b) a multimetal sample (5 mL) containing a broad range of elements (Li, B, Al, Ti, V, Cr, Mn, Fe, Co, Ni, Cu, Zn, Ga, As, Se, Zr, Mo, Ru, Pd, Ag, Cd, In, Sn, Sb, Te, Pt, Au, Hg, Tl, Pb, and Bi). Concentrations were determined by ICP-MS using rhodium as an internal standard. The extraction coefficient (K), defined as the ratio of the metal concentration in the organic phase to that in the aqueous phase at equilibrium, was calculated according to (eq 1). All experiments were performed at 25 °C, either at pH 2 (adjusted with nitric acid) or in a buffered pH 6 solution.

### General Procedure for the Selective Extraction of REE, Thorium, and Uranium (Figure 7)

The selected analytes (Th, Sc, Lu, and Yb, as nitrate salts) were dissolved in an aqueous dihydrogen/monohydrogen phosphate buffer solution at pH 6.0 to prepare sample **A** (5 mL, 0.1 M concentration per element). The extractant—5 mL of a 0.3 M solution of compound **4** in [CYPHOS]­[NTf_2_] (**5**)—was then added, and the mixture was stirred for 12–13 min using a vortex mixer. After the phases were allowed to settle and separate, the metal ions were successfully extracted into the ionic liquid phase (solution **B**), leaving the aqueous phase (solution **A**) essentially metal-free. ICP-MS analysis confirmed an extraction yield of >99.5%, with a residual metal content in the aqueous phase of <0.05%.

### General Procedure (Stripping) for the Liberation of the Extracted Metal and Recuperation of the Extraction System (**4** in **5**) (Figure 8)

To strip the corresponding metal from solution **B** and regenerate the extraction system (**4** in **5**), 5 mL of an aqueous nitric acid solution at pH 0.5 (solution **C**) was added to 5 mL of the metal-saturated IL phase. The mixture was agitated using a vortex mixer for 7 min and then allowed to stand to ensure complete phase separation. The metal was back-extracted into the aqueous phase (solution **C**), effectively recovering the extraction system (solution **B**). A detailed scheme of this recovery process is illustrated in Figure 8. ICP-MS analysis confirmed a metal recovery yield exceeding 98%, demonstrating that the regenerated extraction system remains viable for subsequent extraction cycles.

## Supplementary Material



## Data Availability

NMR Spectra of all new compounds, kinetics of a ternary component extraction survey, and DFT models with their Cartesian coordinates are included in the Supporting Information. “This material is available free of charge via the Internet at http://pubs.acs.org.”

## Notes

Graphics of the extractions, titration experiments, NMR spectra of all characterized compounds and computational methods and Cartesian coordinates are detailed (PDF)
