# Supplementary material for: Modulation of TSIL-Based Ciprofloxacin Structures with pH for the Selective Extraction of Rare Earth Metals, Uranium, Thorium, Titanium, and Zinc
Source: ACS Omega. 2026 Mar 17;11(12):19588–600. doi: 10.1021/acsomega.5c13377 (PMC13044669; doi:10.1021/acsomega.5c13377)
Supplement: Supplementary file 1 [file ao5c13377_si_001.pdf]

Modulation of TSIL based ciprofloxacine structures with pH for the selective extraction of rare earth metals, uranium, thorium titanium and zinc

David Lledó[a], Guillermo Grindlay[b], H. Q. Nimal Gunaratne[c], Abel de Cózar[d,e], Ana Sirvent*[f], and José M. Sansano*[f]

[a] *Medalchemy, S. L. Ancha de Castelar, 46-48, entlo. A. San Vicente del Raspeig, 03690-Alicante (Spain).*

[b] *Department of Analytical Chemistry, Nutrition and Food Sciences, University of Alicante, PO Box 99, 03080 Alicante, Spain.*

[c] *The QUILL Research Centre, School of Chemistry and Chemical Engineering, the Queen's University of Belfast, Stranmillis Road, Belfast, Northern Ireland, BT9 5AG, UK.*

[d] *Departamento de Química Orgánica I/Kimika Organikoa I Saila, Facultad de Química/Kimika Fakultatea, Universidad del País Vasco/Euskal Herriko Unibertsitatea (UPV/EHU) and Donostia International Physics Center (DIPC), and Centro de Innovación en Química Avanzada (ORFEO-CINQA) P. K, 1072, 20018 San Sebastián-Donostia, Spain*

[e] *Ikerbasque, Basque Foundation for Science, Plaza Euskadi 5, 48009, Bilbao, Spain*

[f] *Departamento de Química Orgánica, Centro de Innovación en Química Avanzada (ORFEO-CINQA) and Institute of Organic Synthesis. Universidad de Alicante. Ctra. Alicante-San Vicente s/n, 03080-Alicante, Spain.*

*Email: ana.sirvent@ua.es

*Email: jmsansano@ua.es

TABLE OF CONTENTS

1. Extractions of REEs, uranium and thorium by IL systems S2

2. Titration experiments run by ^{1}H NMR.. S11

3. ^{1}H ^{13}C and ^{19}F NMR spectra S14

4. Computational methods and Cartesian coordinates S17

5. References S36

1. Extractions of REEs, uranium and thorium by IL systems

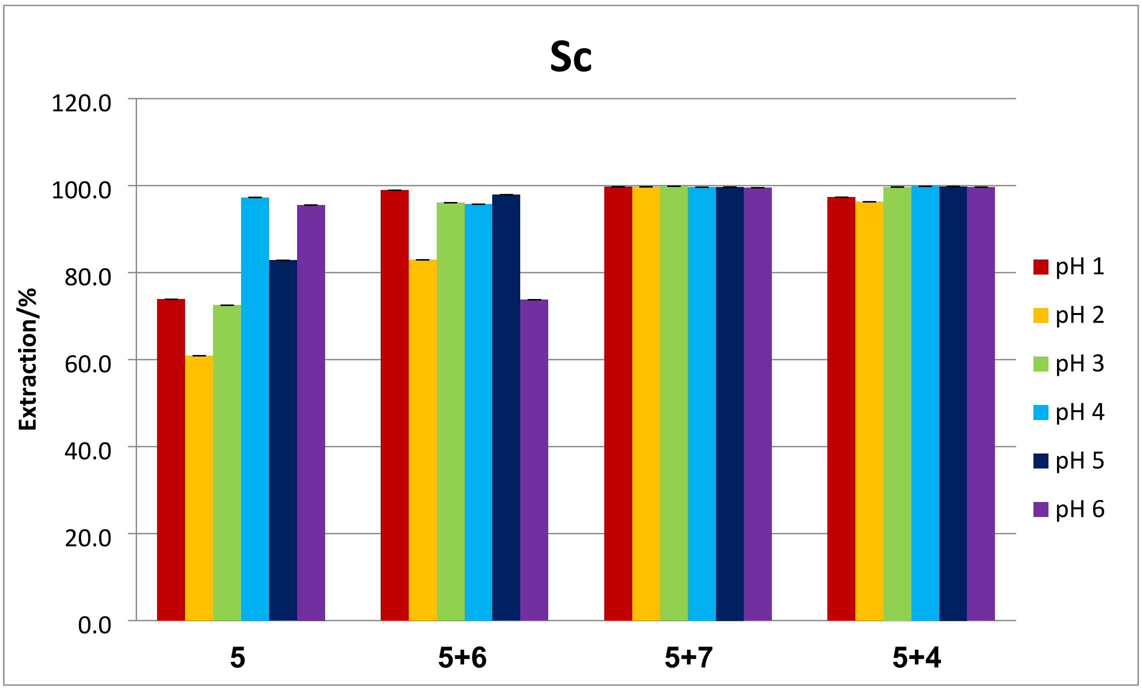

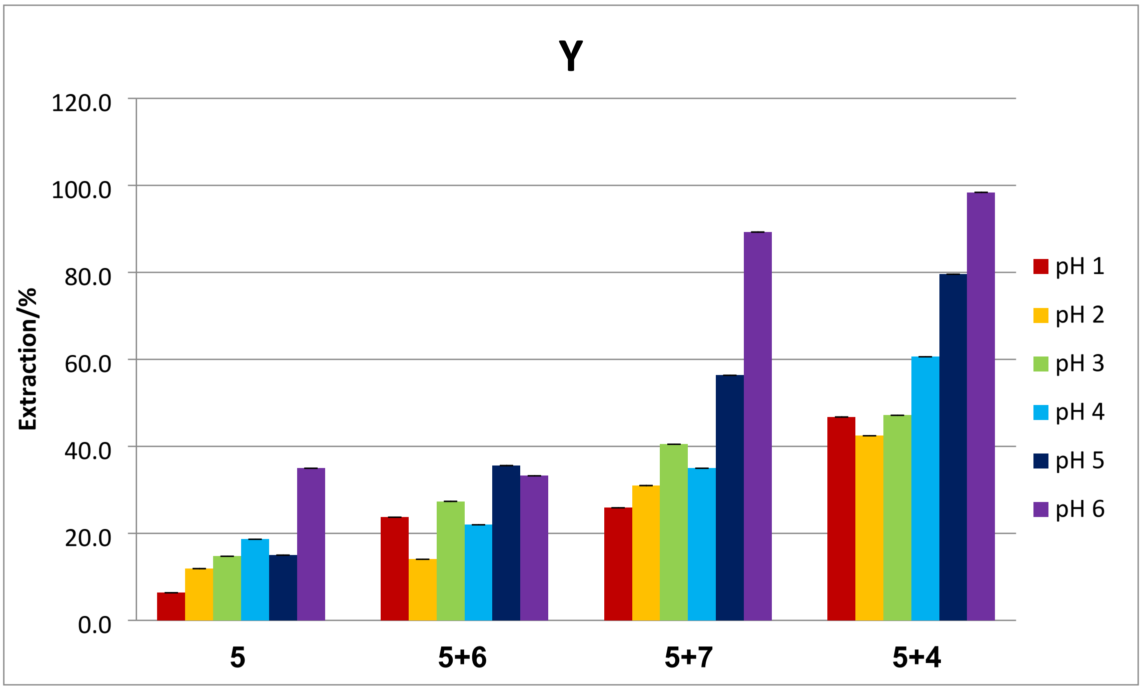

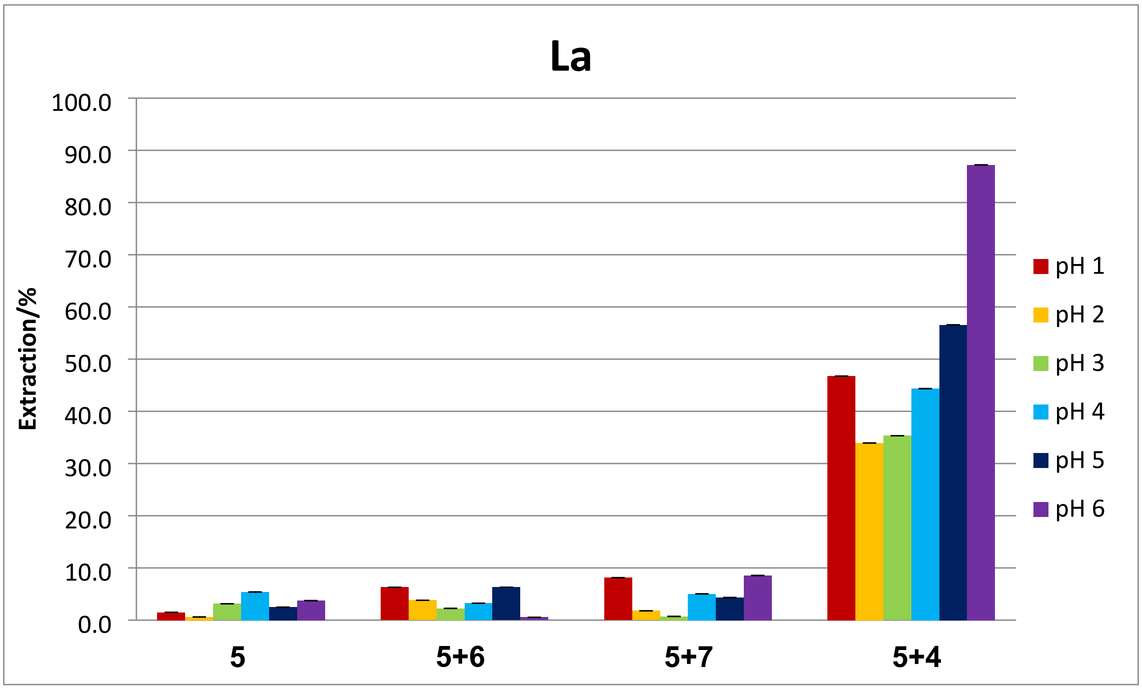
La
Extraction/%
100.0
90.0
80.0
70.0
60.0
50.0
40.0
30.0
20.0
10.0
0.0
5
5+6
5+7
5+4
pH 1
pH 2
pH 3
pH 4
pH 5
pH 6

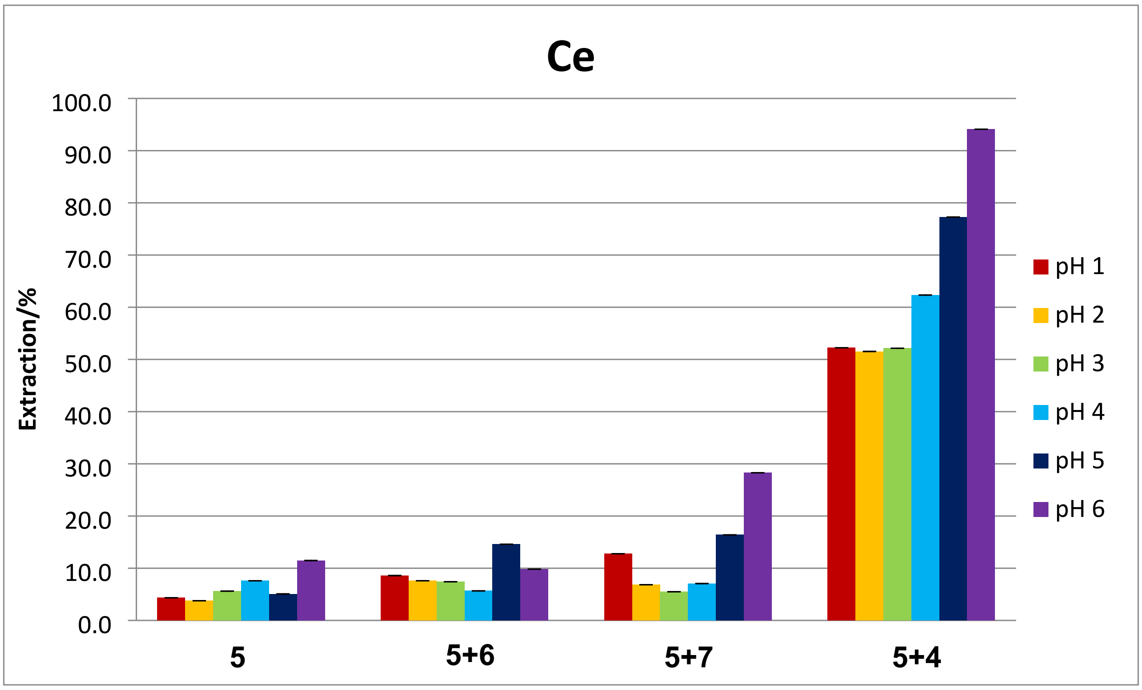
Ce
Extraction/%
100.0
90.0
80.0
70.0
60.0
50.0
40.0
30.0
20.0
10.0
0.0
5
5+6
5+7
5+4
pH 1
pH 2
pH 3
pH 4
pH 5
pH 6

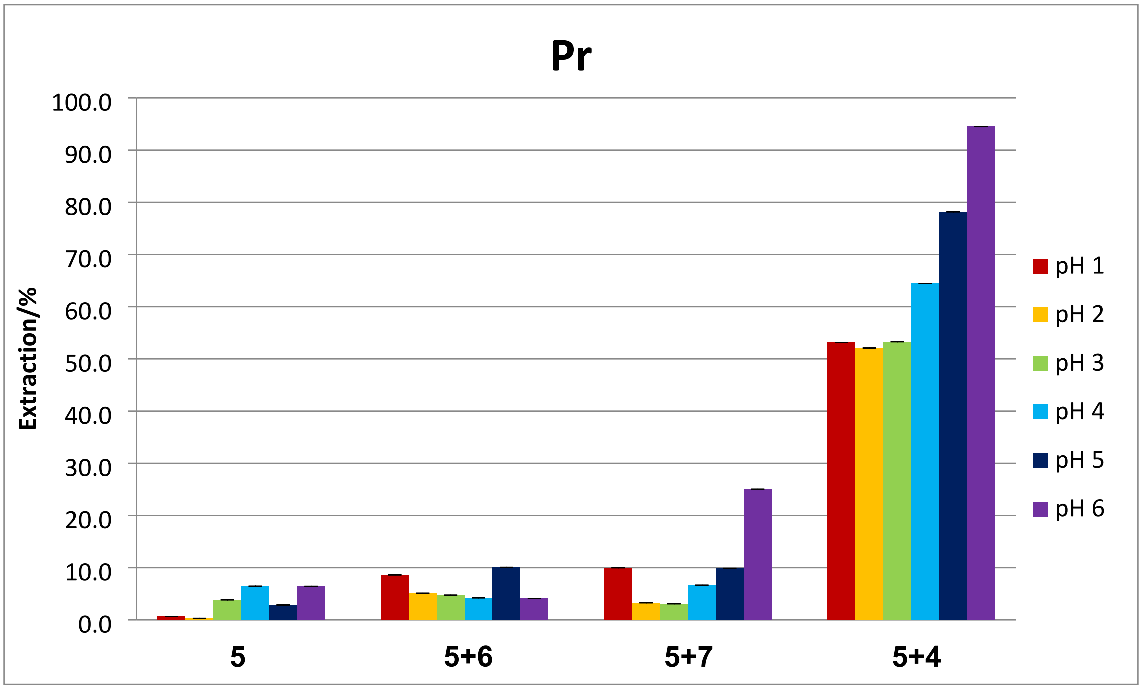
Pr
Extraction/%
100.0
90.0
80.0
70.0
60.0
50.0
40.0
30.0
20.0
10.0
0.0
5
5+6
5+7
5+4
pH 1
pH 2
pH 3
pH 4
pH 5
pH 6

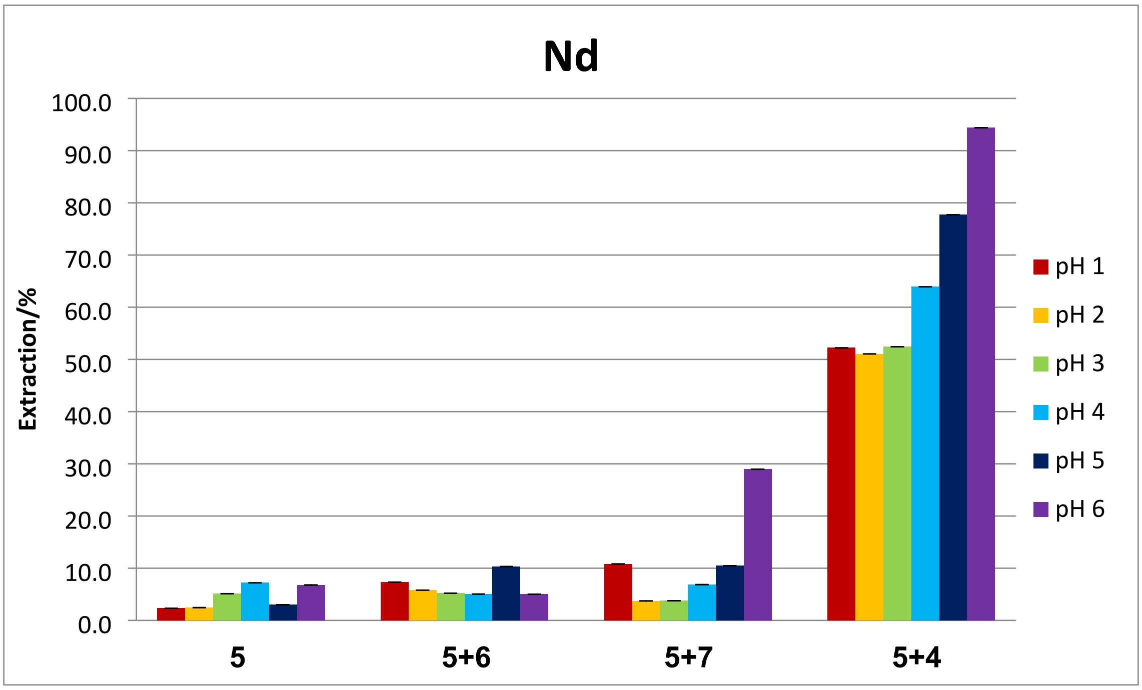
Nd
Extraction/%
100.0
90.0
80.0
70.0
60.0
50.0
40.0
30.0
20.0
10.0
0.0
5
5+6
5+7
5+4
pH 1
pH 2
pH 3
pH 4
pH 5
pH 6

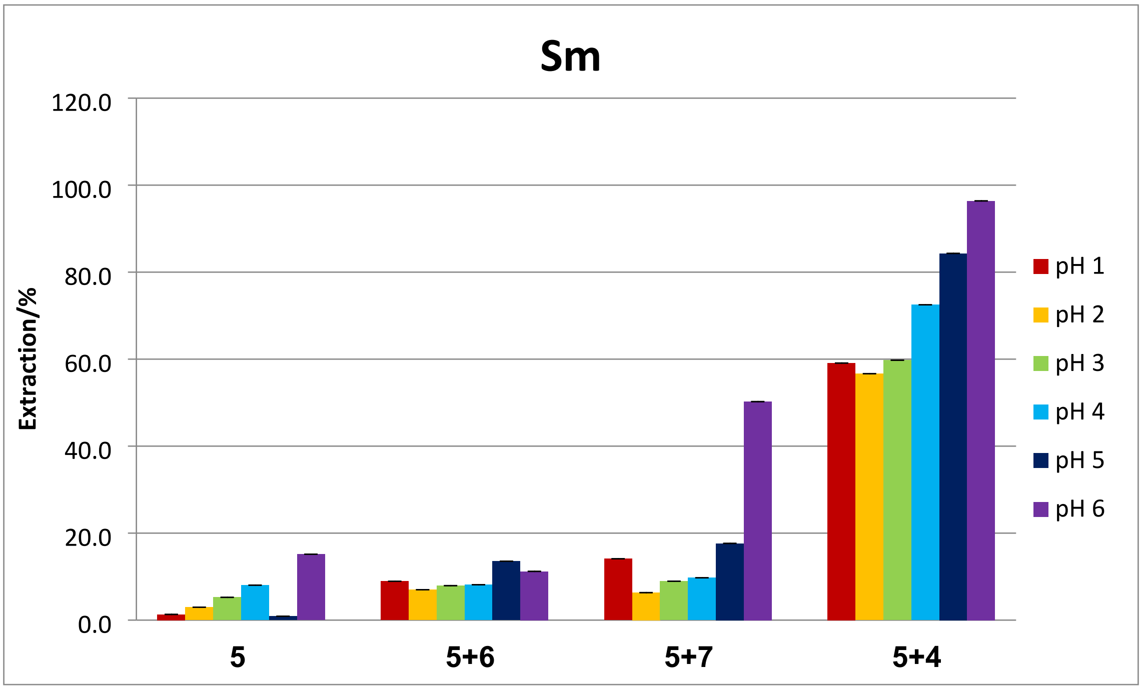
Sm
Extraction/%
120.0
100.0
80.0
60.0
40.0
20.0
0.0
5
5+6
5+7
5+4
pH 1
pH 2
pH 3
pH 4
pH 5
pH 6

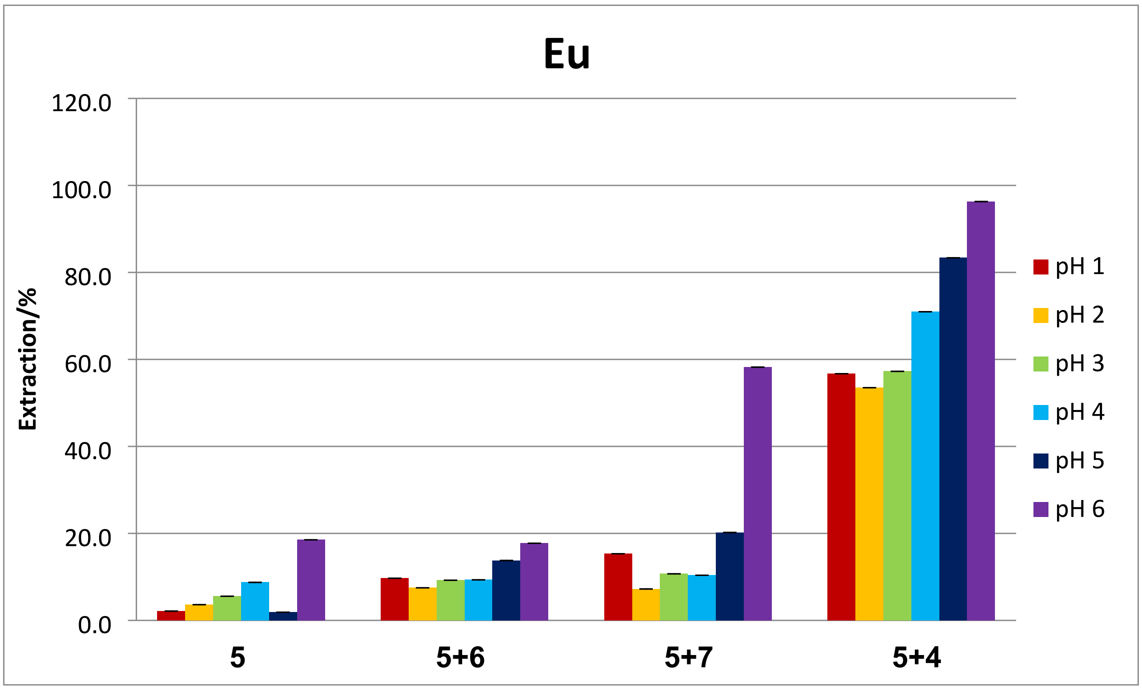
Eu
Extraction/%
120.0
100.0
80.0
60.0
40.0
20.0
0.0
5
5+6
5+7
5+4
pH 1
pH 2
pH 3
pH 4
pH 5
pH 6

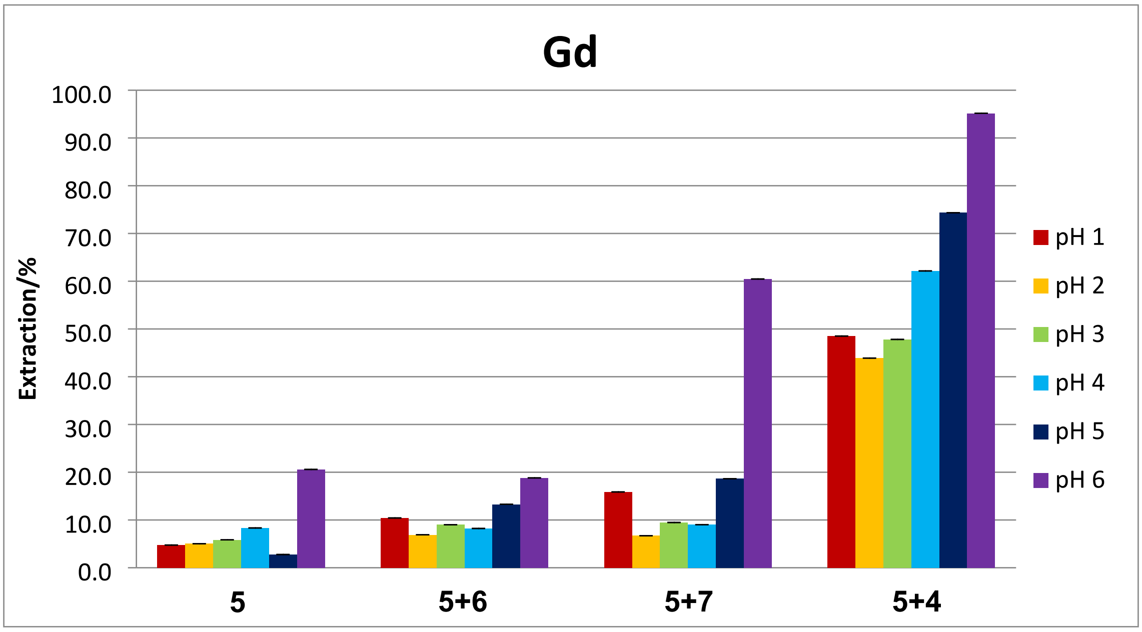
Gd
100.0
90.0
80.0
70.0
60.0
50.0
40.0
30.0
20.0
10.0
0.0
Extraction/%
5
5+6
5+7
5+4
pH 1
pH 2
pH 3
pH 4
pH 5
pH 6

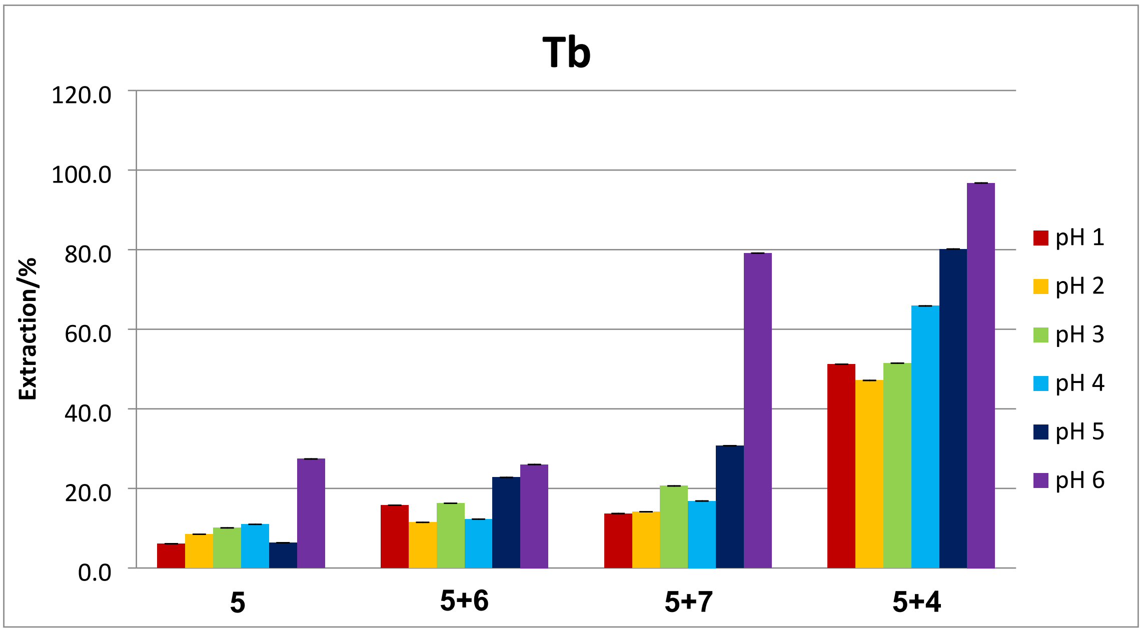
Tb
120.0
100.0
80.0
60.0
40.0
20.0
0.0
Extraction/%
5
5+6
5+7
5+4
pH 1
pH 2
pH 3
pH 4
pH 5
pH 6

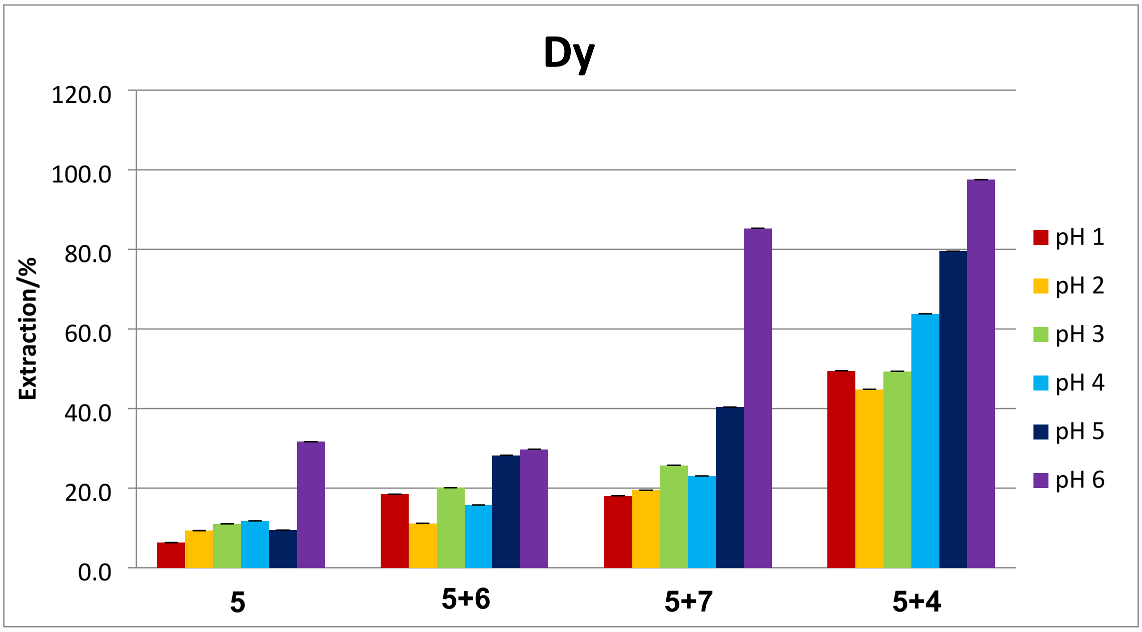
Dy
Extraction/%
120.0
100.0
80.0
60.0
40.0
20.0
0.0
5
5+6
5+7
5+4
pH 1
pH 2
pH 3
pH 4
pH 5
pH 6

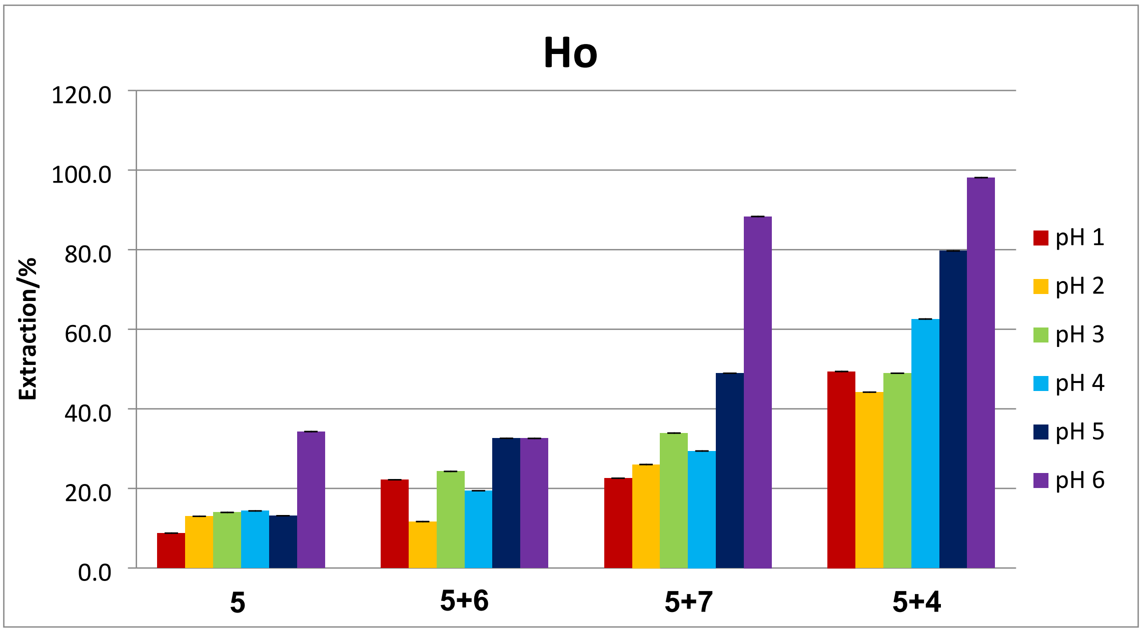
Ho
Extraction/%
120.0
100.0
80.0
60.0
40.0
20.0
0.0
5
5+6
5+7
5+4
pH 1
pH 2
pH 3
pH 4
pH 5
pH 6

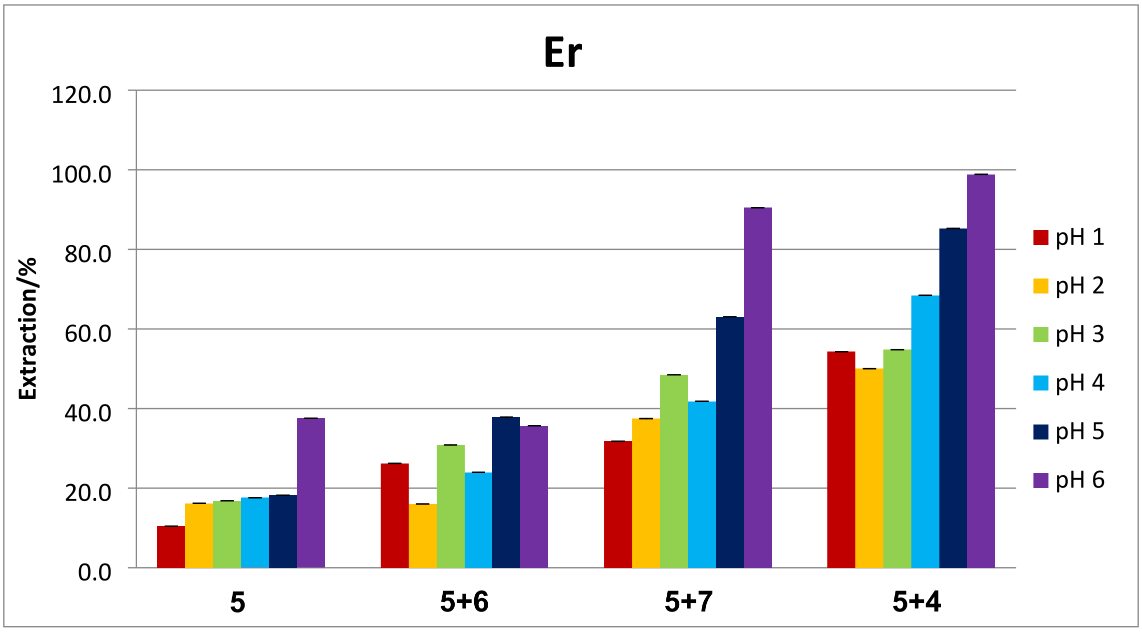
Er
120.0
100.0
80.0
60.0
40.0
20.0
0.0
Extraction/%
5
5+6
5+7
5+4
pH 1
pH 2
pH 3
pH 4
pH 5
pH 6

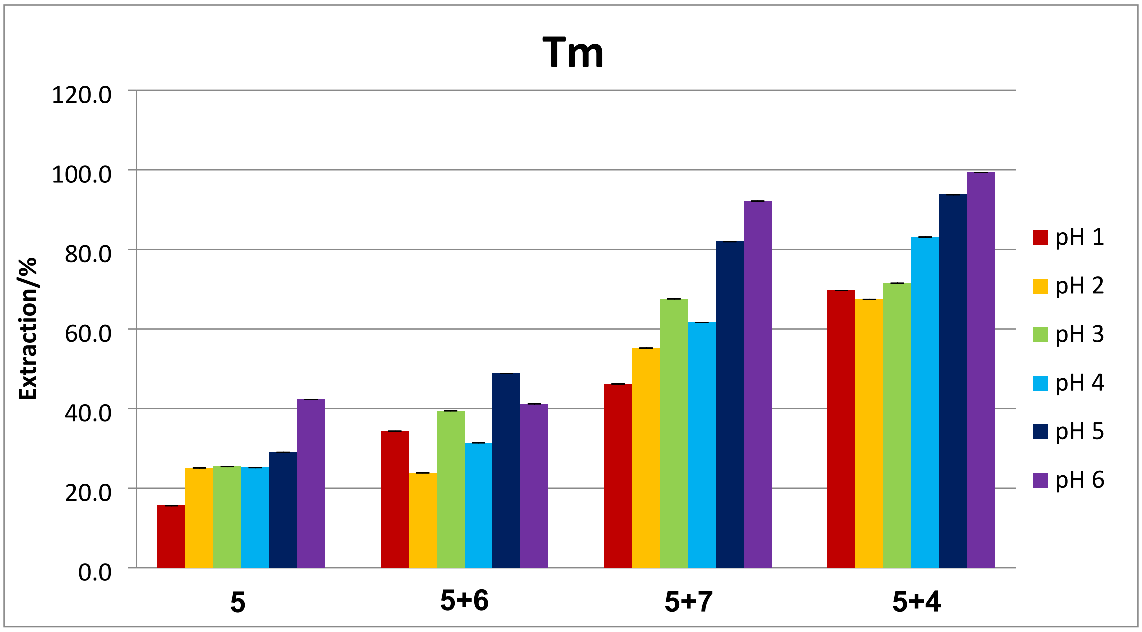
Tm
120.0
100.0
80.0
60.0
40.0
20.0
0.0
Extraction/%
5
5+6
5+7
5+4
pH 1
pH 2
pH 3
pH 4
pH 5
pH 6

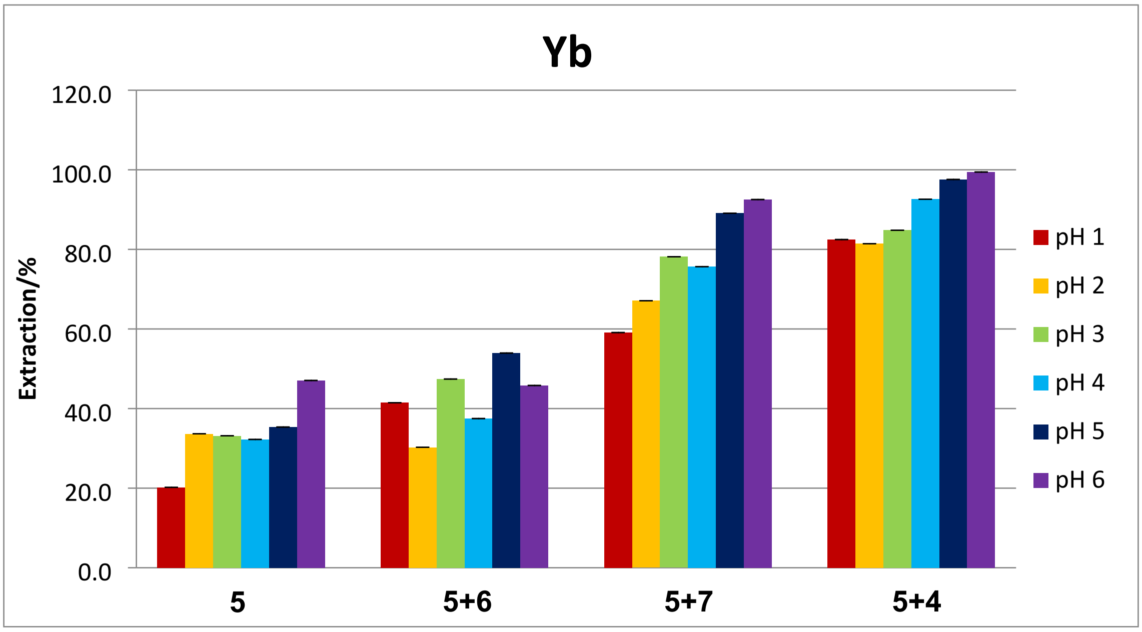
Yb
120.0
100.0
80.0
60.0
40.0
20.0
0.0
Extraction/%
5
5+6
5+7
5+4
pH 1
pH 2
pH 3
pH 4
pH 5
pH 6

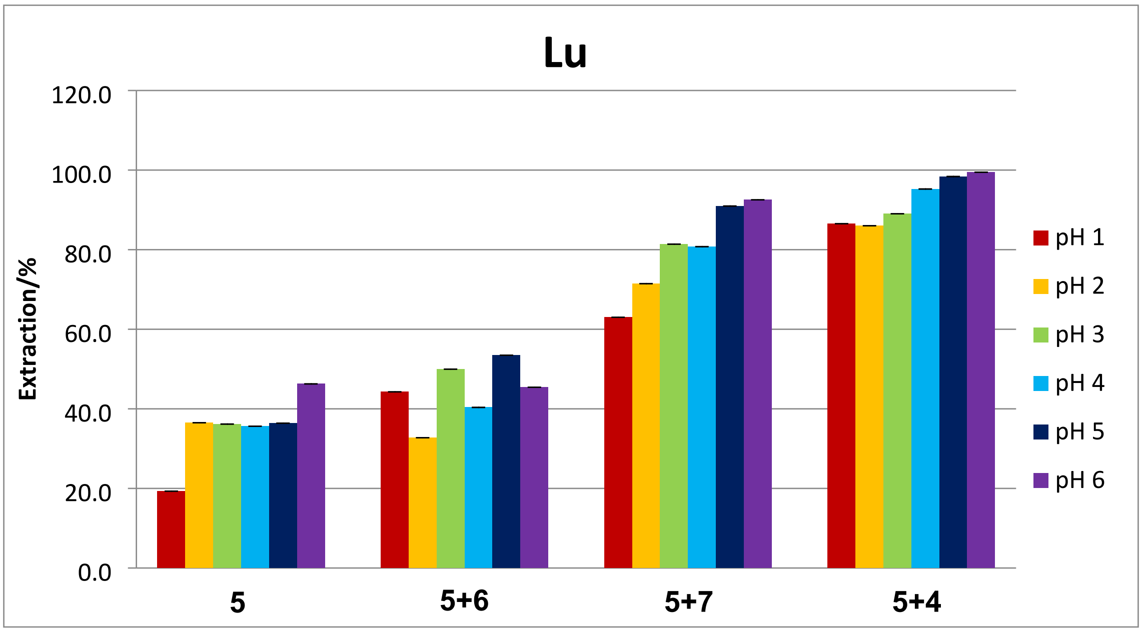
Lu
120.0
100.0
80.0
60.0
40.0
20.0
0.0
Extraction/%
5
5+6
5+7
5+4
pH 1
pH 2
pH 3
pH 4
pH 5
pH 6

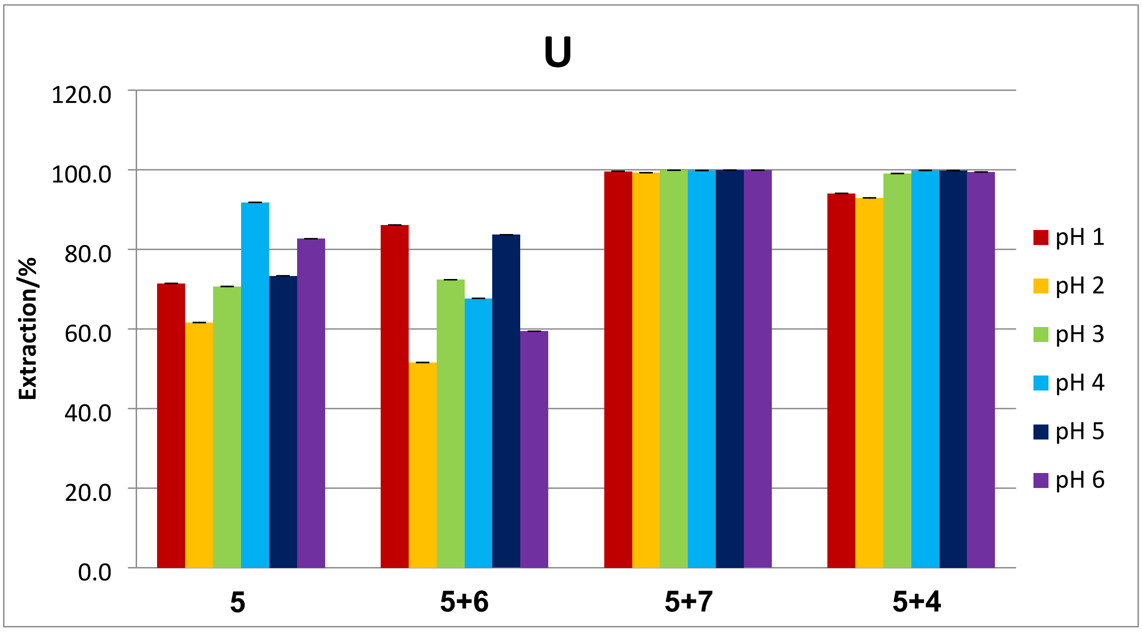

U
120.0
100.0
80.0
60.0
40.0
20.0
0.0
Extraction/%
5
5+6
5+7
5+4
pH 1
pH 2
pH 3
pH 4
pH 5
pH 6

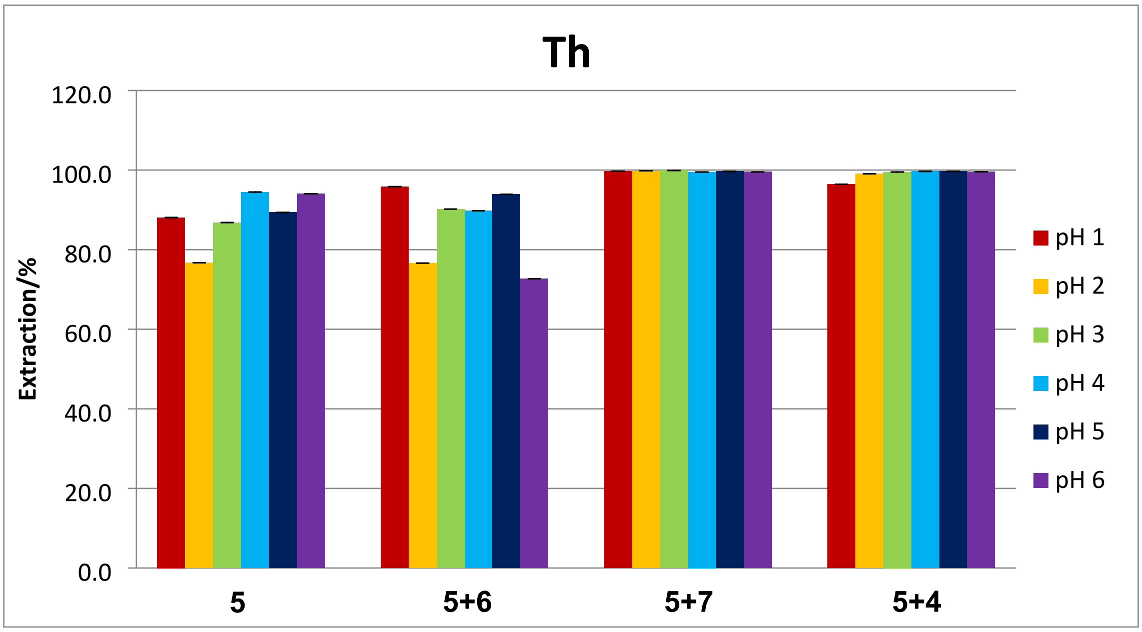

Th
120.0
100.0
80.0
60.0
40.0
20.0
0.0
Extraction/%
5
5+6
5+7
5+4
pH 1
pH 2
pH 3
pH 4
pH 5
pH 6

2. Titration experiments run by ^{1}H NMR

Yb

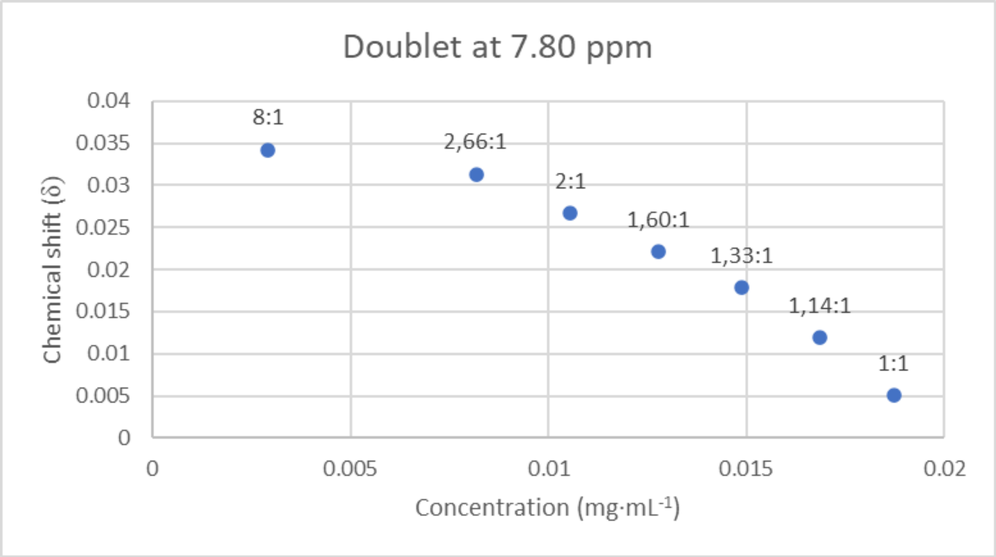

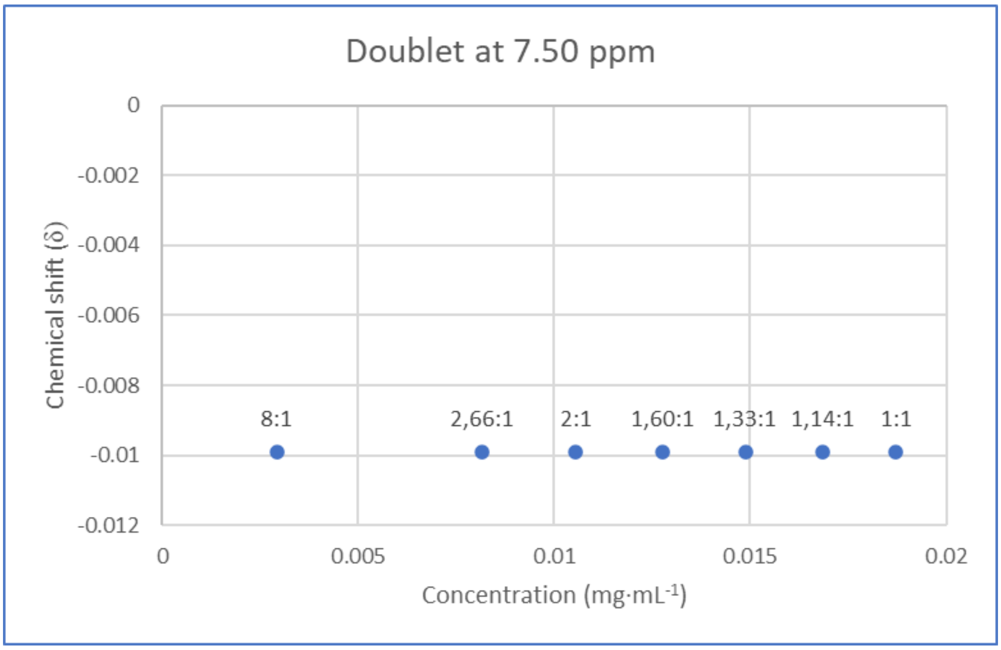

Sc

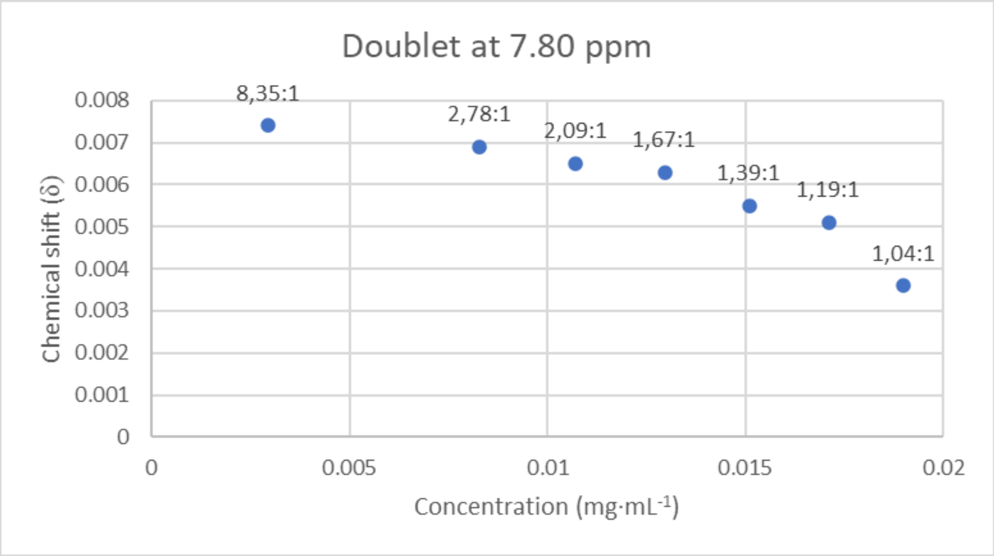

Doublet at 7.80 ppm
Chemical shift (δ)
Concentration (mg·mL-1)
0.008
0.007
0.006
0.005
0.004
0.003
0.002
0.001
0
0.005
0.01
0.015
0.02
8,35:1
2,78:1
2,09:1
1,67:1
1,39:1
1,19:1
1,04:1

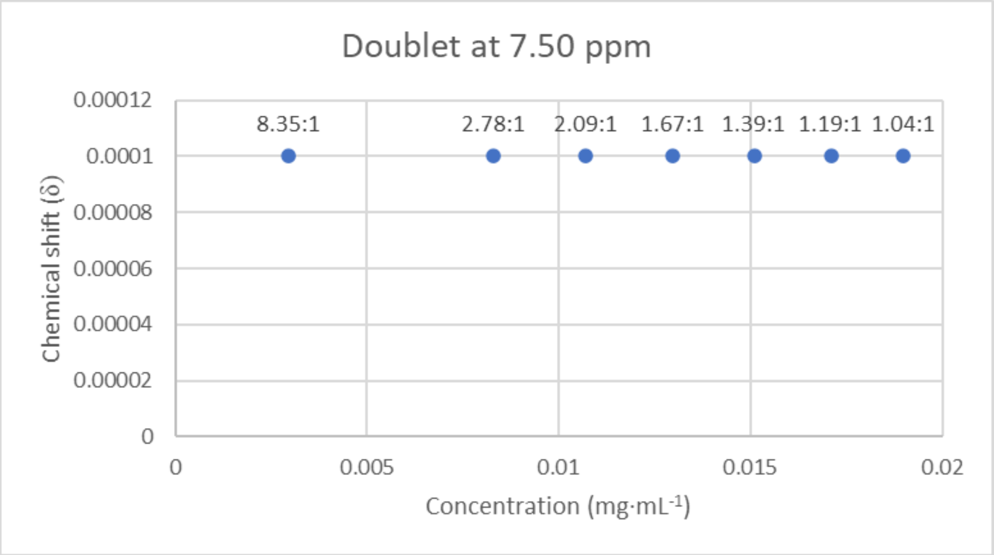

Doublet at 7.50 ppm
Chemical shift (δ)
Concentration (mg·mL-1)
0.00012
0.0001
0.00008
0.00006
0.00004
0.00002
0
0.005
0.01
0.015
0.02
8.35:1
2.78:1
2.09:1
1.67:1
1.39:1
1.19:1
1.04:1

Th

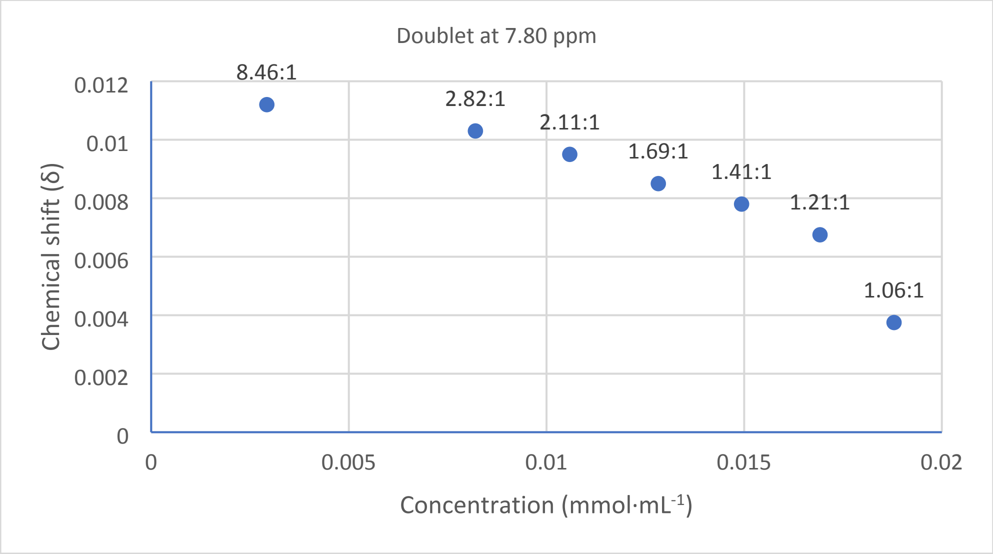
Doublet at 7.80 ppm
Chemical shift (δ)
0.012
0.01
0.008
0.006
0.004
0.002
0
0
0.005
0.01
0.015
0.02
Concentration (mmol·mL-1)
8.46:1
2.82:1
2.11:1
1.69:1
1.41:1
1.21:1
1.06:1

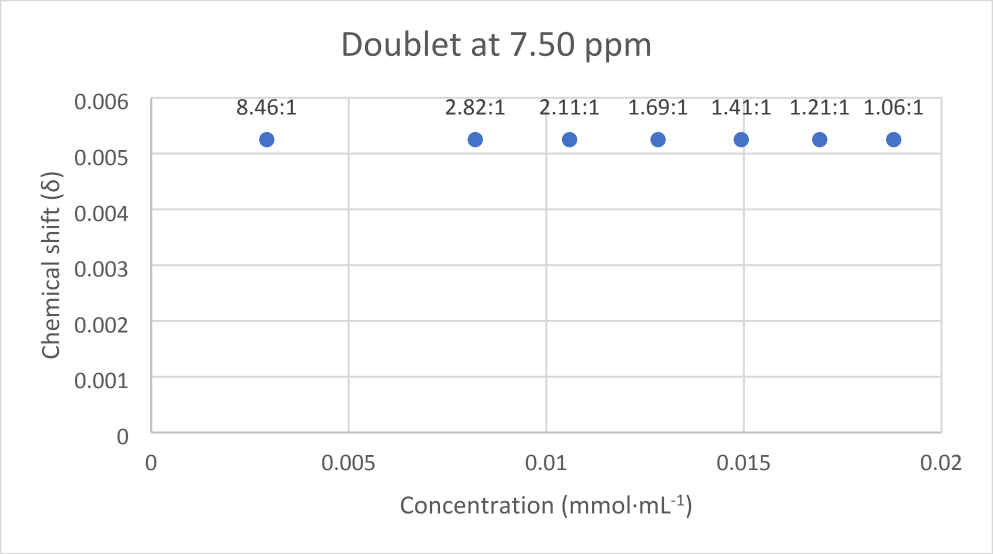
Doublet at 7.50 ppm
Chemical shift (δ)
0.006
0.005
0.004
0.003
0.002
0.001
0
0
0.005
0.01
0.015
0.02
Concentration (mmol·mL-1)
8.46:1
2.82:1
2.11:1
1.69:1
1.41:1
1.21:1
1.06:1

3. ^{1}H ^{13}C and ^{19}F NMR spectra

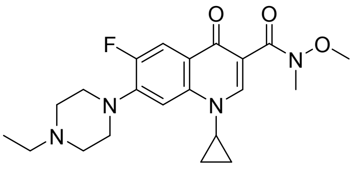

3

(^{1}H NMR, 400 MHz, $CDCl_3$)

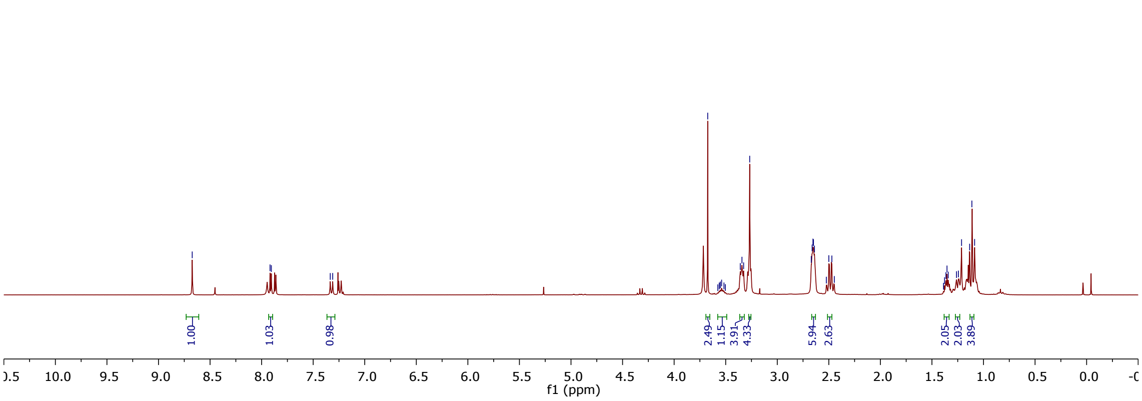

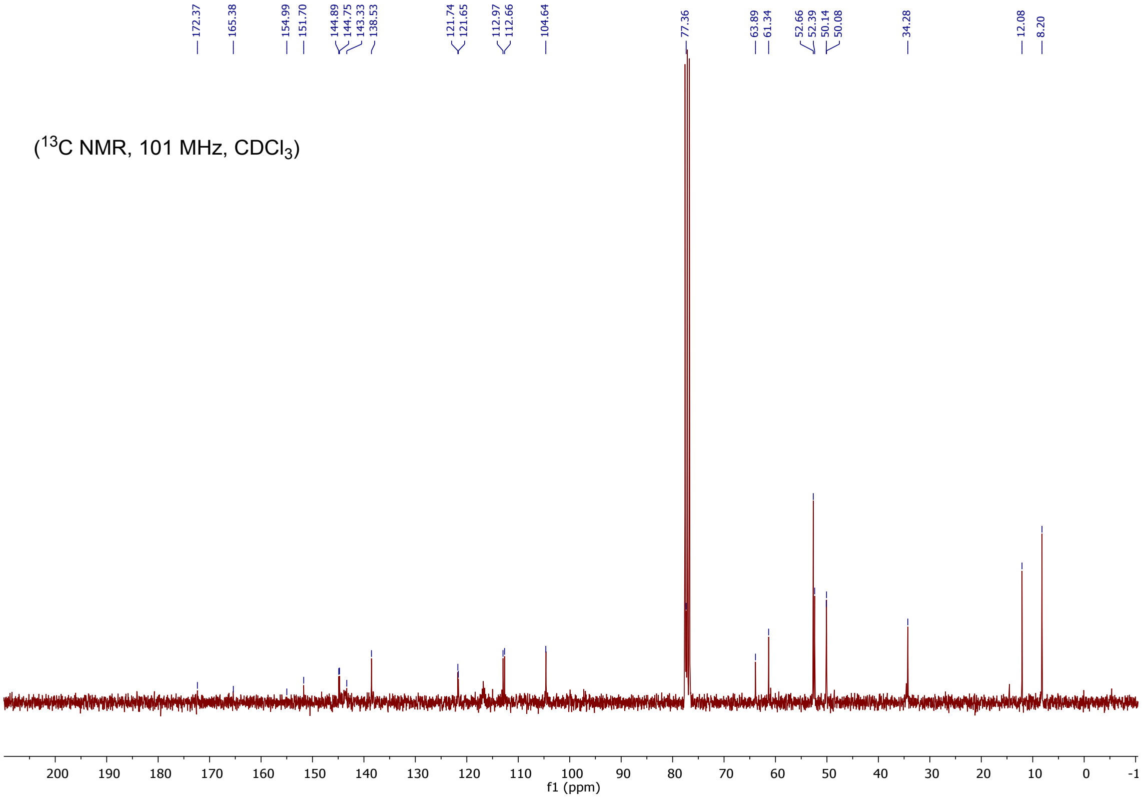

(^{13}C NMR, 101 MHz, $CDCl_3$)

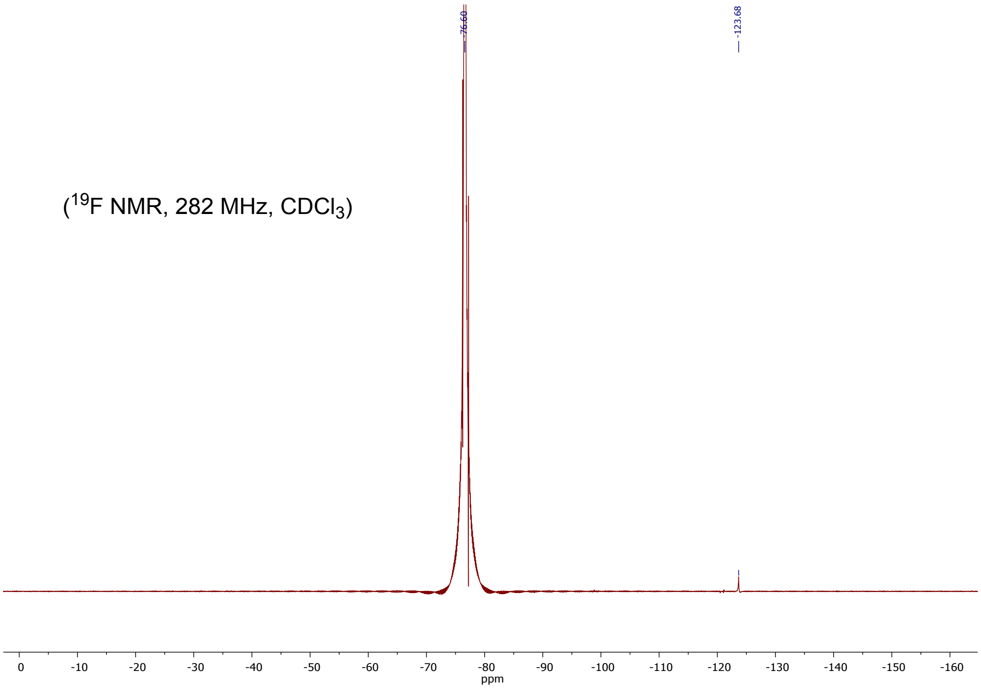
-76.60
-123.68
(19F NMR, 282 MHz, CDCl3)
0 -10 -20 -30 -40 -50 -60 -70 -80 -90 -100 -110 -120 -130 -140 -150 -160
ppm

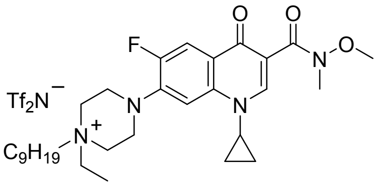
F
O
O
N
O
Tf2N
N
N
+
C9H19
N

4

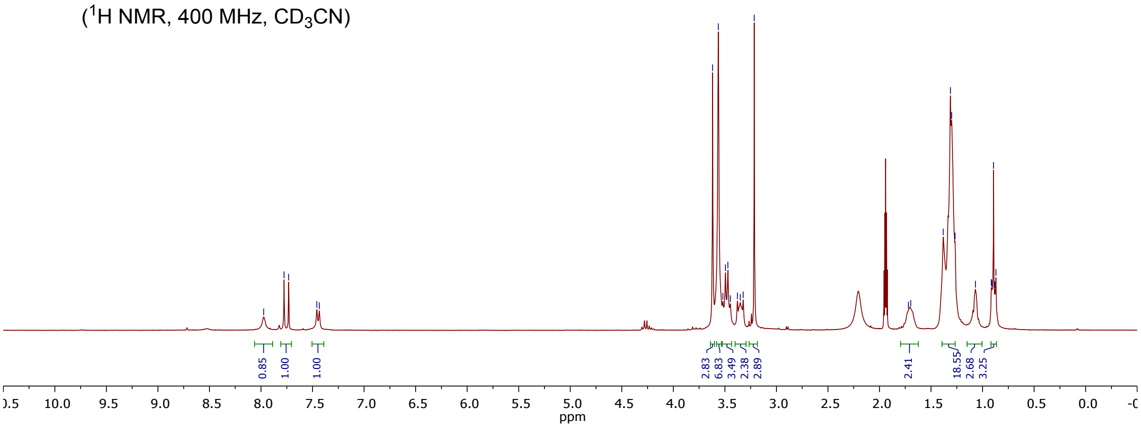
(1H NMR, 400 MHz, CD3CN)
7.97
7.78
7.73
7.46
7.43
3.62
3.56
3.52
3.49
3.47
3.45
3.38
3.35
3.32
3.21
1.72
1.70
1.38
1.31
1.30
1.27
1.07
0.92
0.91
0.89
0.88
0.87
0.85
1.00
1.00
2.83
6.83
3.49
2.38
2.89
2.41
18.55
2.68
3.25
10.0 9.5 9.0 8.5 8.0 7.5 7.0 6.5 6.0 5.5 5.0 4.5 4.0 3.5 3.0 2.5 2.0 1.5 1.0 0.5 0.0
ppm

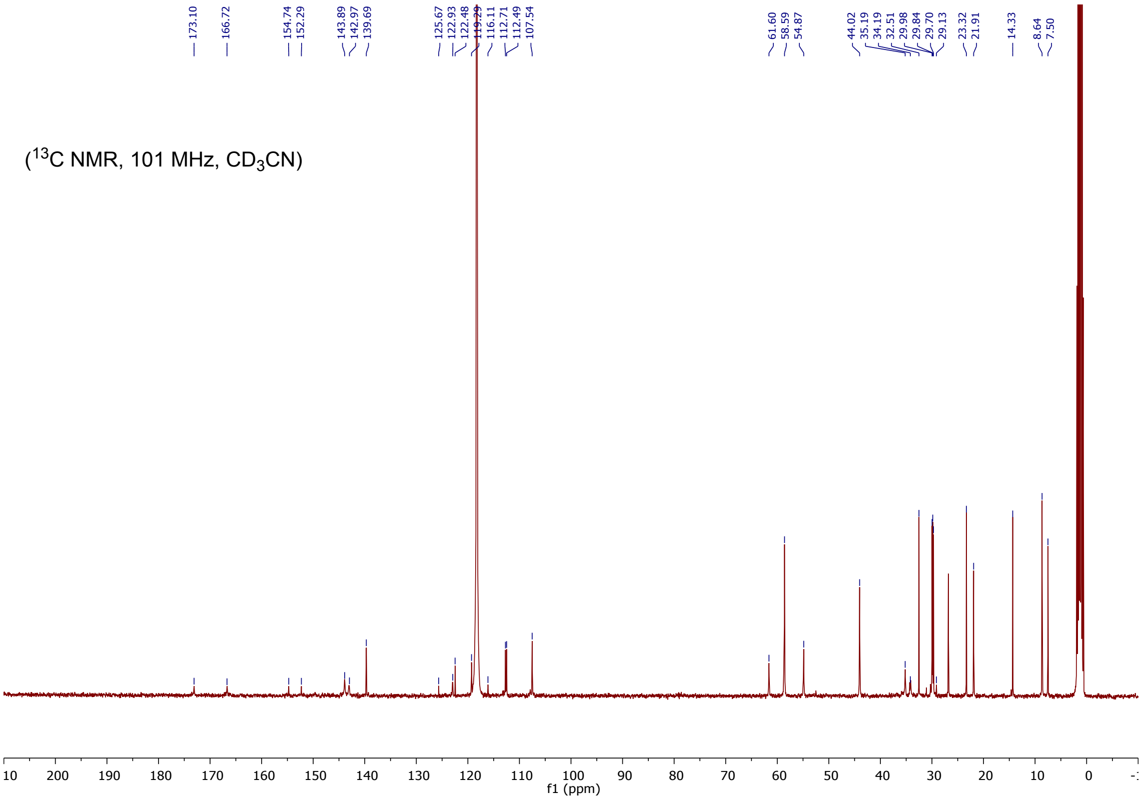
173.10
166.72
154.74
152.29
143.89
142.97
139.69
125.67
122.93
122.48
119.29
116.11
112.71
112.49
107.54
61.60
58.59
54.87
44.02
35.19
34.19
32.51
29.98
29.84
29.70
29.13
23.32
21.91
14.33
8.64
7.50
(^{13}C NMR, 101 MHz, CD_3CN)
f1 (ppm)

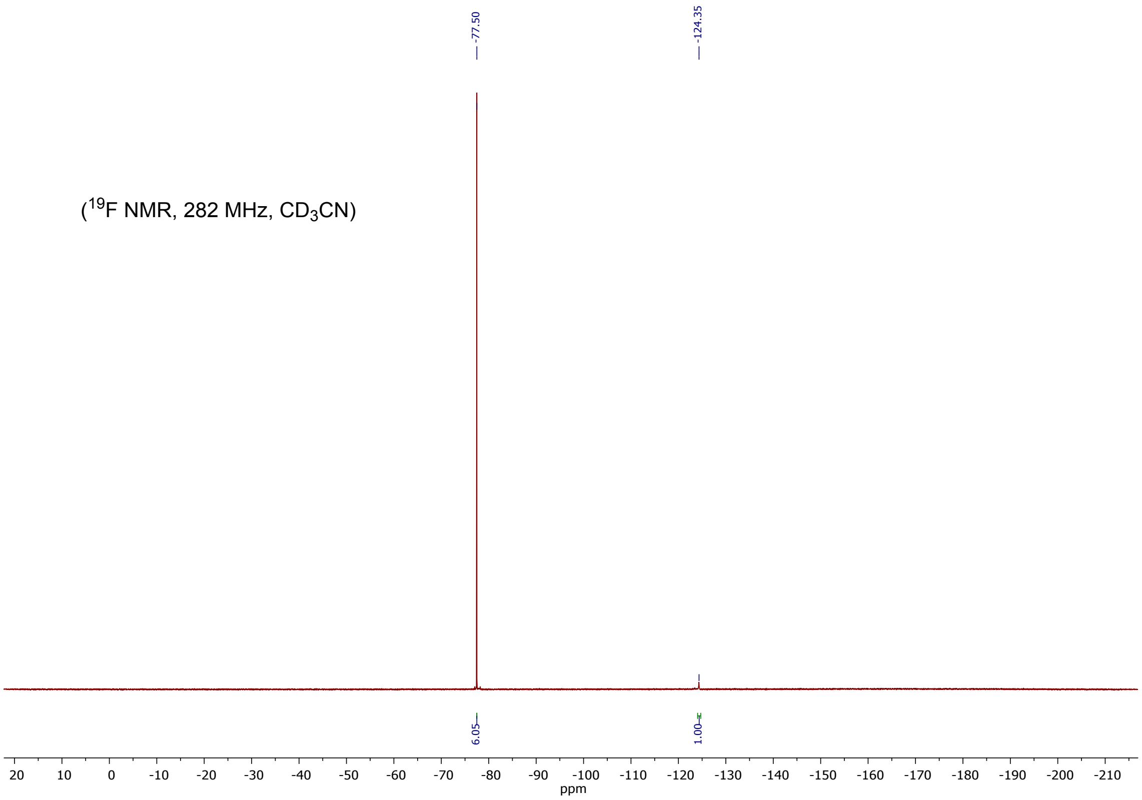
-77.50
-124.35
(^{19}F NMR, 282 MHz, CD_3CN)
6.05
1.00
ppm

4. Computational methods and Cartesian coordinates.

Theoretical calculations have been carried out by using the GAUSSIAN 16[1] programs. Optimizations were computed within the DFT framework[2] using the Truhlard's metayhybrid M06[3] functional in combination with 6-31g(d) basis set for C, H, N, Fand O atoms and Stuttgart/Dresden[4] effective core potential SDD for La and Lu atoms as implemented in GAUSSIAN 16. Dispersion corrections are included by means of Grimme's D3 model.[5] All the stationary points were characterized by harmonic vibrational analysis. Local minima showed positive definite Hessians and thermal corrections were not scaled. Solvent effects were estimated by the polarization continuum model[6] (PCM) method within the self-consistent reaction field (SCRF) approach. [7] All SCRF-PCM calculations were performed using water (ε=78.3553) as model solvent considering Solvent Accessible Surfaces (SAS).

Table S1. Total electronic energies[a] (E, in a.u.), zero point correction of the energy[b] (ZPC), thermal corrections to Gibbs free energies[b] (TCGFE, in a.u.), and number of imaginary frequencies (NIMAG) of all stationary points discussed in the main text and in the Supporting Information.

Structure	E	ZPC	TCGFE	NIMAG
**4**-cation	-1557.257328	0.632328	0.565434	0
H_2O	-76.374994	0.021519	0.003862	0
**(**$NO_2)_2$·La$(H_2O)_6^+$	-1454.320974	0.183409	0.126916	0
**(**$NO_2)_2$·La$(4)_3^{+4}$	-5667.831960	1.941105	1.784552	0
**(**$NO_2)_2$·Lu$(H_2O)_5^+$	-2179.070426	0.160830	0.110773	0
**(**$NO_2)_2$·Lu$(4)_3^{+4}$	-6468.980518	1.941971	1.784691	0

[a]Computed at M06–D3(PCM)/6-31G(d)&SDD level. [b]Computed at 298.15 K.

Cartesian coordinates (optimized at the M06–D3(PCM)/6-31G(d)&SDD level) of all the stationary points collected in the main text

4-cation

Center Number	Atomic Number	Atomic Type	Coordinates (Angstroms) X	Y	Z
1	7	0	-2.076963	1.458467	-0.653381
2	6	0	-1.205972	0.422848	-0.316101

3	6	0	-1.725886	-0.859410	-0.079342
4	6	0	-3.171583	-1.167468	-0.201416
5	6	0	-3.990629	0.001224	-0.507041
6	6	0	-3.408196	1.200109	-0.773295
7	6	0	-0.849941	-1.907321	0.237377
8	6	0	0.493350	-1.671329	0.314075
9	6	0	1.041147	-0.387208	0.109267
10	6	0	0.180657	0.650207	-0.220213
11	7	0	2.433018	-0.236734	0.158754
12	6	0	3.040011	0.922095	-0.463023
13	6	0	4.566146	0.817274	-0.340236
14	7	0	5.051282	-0.609020	-0.394163
15	6	0	4.451825	-1.394126	0.773177
16	6	0	3.191065	-0.717946	1.302900
17	6	0	4.608208	-1.214564	-1.724832
18	6	0	5.240604	-2.553113	-2.066069
19	6	0	6.566625	-0.650334	-0.314665
20	6	0	7.196845	0.144598	0.834492
21	6	0	7.707580	1.537902	0.452837
22	6	0	8.382004	2.227367	1.635401
23	9	0	1.349475	-2.685553	0.561206
24	8	0	-3.594697	-2.309649	-0.096720
25	6	0	-5.467850	-0.076596	-0.725715
26	8	0	-6.011127	0.392753	-1.719544
27	6	0	-1.566182	2.769906	-0.978390
28	6	0	-2.304690	3.973472	-0.478230
29	6	0	-0.979661	3.610866	0.120313
30	7	0	-6.200475	-0.658515	0.257614
31	8	0	-5.580370	-1.067662	1.418439
32	6	0	-5.666254	-0.059547	2.419813
33	6	0	-7.632906	-0.884764	0.135128
34	6	0	-8.467791	0.384564	0.288780
35	1	0	0.574163	1.642029	-0.398713
36	1	0	-1.256338	-2.902532	0.381969

37	1	0	-4.013338	2.038314	-1.102939
38	1	0	2.751177	1.873171	0.012192
39	1	0	2.727545	0.966444	-1.511307
40	1	0	3.433090	0.102654	1.993738
41	1	0	2.616187	-1.453268	1.861776
42	1	0	4.904973	1.225419	0.611800
43	1	0	5.063551	1.364589	-1.143621
44	1	0	4.205102	-2.384351	0.390024
45	1	0	5.214719	-1.490354	1.546266
46	1	0	4.858271	-0.468634	-2.484404
47	1	0	3.523723	-1.302795	-1.653016
48	1	0	4.742437	-2.922839	-2.965324
49	1	0	6.305086	-2.474236	-2.290813
50	1	0	5.096001	-3.305258	-1.286271
51	1	0	6.813135	-1.710132	-0.236754
52	1	0	6.930855	-0.287353	-1.281585
53	1	0	8.048306	-0.442323	1.194759
54	1	0	6.515280	0.212827	1.692682
55	1	0	8.420639	1.435256	-0.373477
56	1	0	6.892715	2.168202	0.079416
57	1	0	8.762603	3.210268	1.349047
58	1	0	7.679782	2.366439	2.463505
59	1	0	9.223688	1.633662	2.004960
60	1	0	-1.107732	2.830875	-1.962631
61	1	0	-3.183564	3.809107	0.135650
62	1	0	-2.364321	4.827894	-1.141727
63	1	0	-0.113472	4.219313	-0.115800
64	1	0	-0.989668	3.184531	1.118070
65	1	0	-5.062591	-0.424754	3.251958
66	1	0	-5.259328	0.887290	2.047884
67	1	0	-6.700018	0.084092	2.752033
68	1	0	-7.881424	-1.632966	0.891834
69	1	0	-7.808168	-1.323710	-0.851633
70	1	0	-9.528769	0.152950	0.157071

71	1	0	-8.337449	0.835877	1.276013
72	1	0	-8.171748	1.109128	-0.472597

H_2O

Center Number	Atomic Number	Atomic Type	Coordinates (Angstroms)		
			X	Y	Z
1	8	0	-0.000000	0.000000	0.118907
2	1	0	0.000000	-0.761151	-0.475628
3	1	0	0.000000	0.761151	-0.475628

($NO_2)_2{\cdot}La(H_2O)_6^+$

Center Number	Atomic Number	Atomic Type	Coordinates (Angstroms)		
			X	Y	Z
1	8	0	1.453292	2.178777	0.666549
2	57	0	-0.028894	0.143376	-0.042759
3	8	0	-0.195496	0.387778	2.500611
4	8	0	2.273026	-0.481929	-0.919836
5	7	0	2.795274	-0.783984	0.202489
6	8	0	3.920051	-1.181156	0.302957
7	8	0	2.033135	-0.633013	1.221157
8	8	0	0.546081	1.855904	-1.909917
9	8	0	0.081035	-0.827091	-2.457615
10	8	0	-0.071135	-2.348723	0.477888
11	8	0	-2.150650	-0.933582	-0.910484
12	7	0	-2.926804	-0.634563	0.060554
13	8	0	-4.072152	-0.980780	0.092589
14	8	0	-1.236697	2.436079	0.078305
15	8	0	-2.393873	0.056478	0.991099
16	1	0	0.862751	2.846704	1.055631
17	1	0	0.664408	1.386793	-2.753311

18	1	0	-0.687101	-1.335745	-2.765577
19	1	0	-0.760825	-2.989024	0.244846
20	1	0	-1.259574	2.942261	-0.750760
21	1	0	0.481936	0.011643	3.084743
22	1	0	0.565928	-2.787793	1.062874
23	1	0	0.874484	-1.371953	-2.593376
24	1	0	1.397379	2.270818	-1.688246
25	1	0	-2.137733	2.408868	0.440729
26	1	0	2.138873	1.992122	1.328077
27	1	0	-1.065801	0.152750	2.863872

($NO_2)_2$·La$(4)_3^{+4}$

Center Number	Atomic Number	Atomic Type	Coordinates (Angstroms)		
			X	Y	Z
1	6	0	7.542056	-2.674794	-1.542998
2	7	0	6.681311	-1.531450	-1.781386
3	6	0	7.252888	-0.459752	-2.612373
4	6	0	8.727158	-0.723280	-2.870486
5	7	0	8.966447	-2.044054	-3.561349
6	6	0	8.171007	-3.135567	-2.850071
7	6	0	5.729879	-1.209042	-0.825016
8	6	0	4.730185	-0.246827	-1.129248
9	6	0	3.773585	0.128182	-0.232034
10	6	0	3.733575	-0.477800	1.038176
11	6	0	4.673879	-1.475741	1.348922
12	6	0	5.673325	-1.825332	0.423294
13	7	0	4.590431	-2.122273	2.582276
14	6	0	3.616999	-1.792547	3.447895
15	6	0	2.699824	-0.795152	3.233705
16	6	0	2.696681	-0.088346	1.979595
17	8	0	1.878281	0.814360	1.695018
18	57	0	-0.595390	0.618653	1.841418

19	8	0	0.284872	0.788568	-0.680143
20	7	0	0.574110	2.013214	-0.583552
21	8	0	1.333862	2.562552	-1.365485
22	6	0	5.450079	-3.254109	2.877856
23	6	0	6.907798	-3.011893	3.141558
24	6	0	5.995927	-3.400327	4.265099
25	6	0	1.634894	-0.518903	4.227435
26	8	0	0.469597	-0.295058	3.865823
27	9	0	4.728802	0.285885	-2.359407
28	6	0	8.491939	-1.928798	-5.005173
29	6	0	8.760281	-3.154975	-5.861621
30	6	0	10.451718	-2.365576	-3.566262
31	6	0	11.116486	-2.411530	-2.184133
32	6	0	11.974736	-1.181817	-1.854760
33	6	0	13.243963	-1.109243	-2.701916
34	8	0	-1.995956	-1.215297	2.673566
35	6	0	-2.419107	-2.151139	1.951977
36	6	0	-3.833733	-2.349176	1.730692
37	6	0	-4.313602	-3.407407	0.945396
38	7	0	-3.405911	-4.315861	0.406042
39	6	0	-2.089703	-4.092790	0.518444
40	6	0	-1.548607	-3.065925	1.259519
41	6	0	-4.743939	-1.383132	2.186849
42	6	0	-6.053540	-1.443147	1.812734
43	6	0	-6.562118	-2.479278	0.978327
44	6	0	-5.674279	-3.498232	0.634592
45	7	0	-7.836943	-2.479689	0.433384
46	6	0	-8.025197	-3.034722	-0.908282
47	6	0	-8.787498	-2.046940	-1.803456
48	7	0	-8.347097	-0.627935	-1.555783
49	6	0	-8.686556	-0.247186	-0.115956
50	6	0	-8.852247	-1.492661	0.759523
51	6	0	-6.841113	-0.548291	-1.811494
52	6	0	-6.247366	0.847922	-1.814239

53	6	0	-9.047979	0.322954	-2.510249
54	6	0	-10.570084	0.175160	-2.605708
55	6	0	-11.067306	-0.677657	-3.777674
56	6	0	-12.591708	-0.690227	-3.848743
57	9	0	-6.860124	-0.431318	2.192718
58	6	0	-0.098132	-2.764606	1.199083
59	8	0	0.299251	-1.597372	1.045173
60	6	0	-3.885611	-5.354063	-0.483644
61	6	0	-3.216862	-6.690752	-0.459238
62	6	0	-4.597956	-6.532684	0.108190
63	7	0	0.781351	-3.790036	1.180873
64	8	0	2.113218	-3.450884	1.008816
65	6	0	2.394306	-3.194618	-0.370720
66	6	0	0.638060	-5.133936	1.741926
67	6	0	0.984355	-6.214509	0.728549
68	8	0	0.055323	2.699452	3.209407
69	7	0	-1.049655	3.278964	3.030671
70	8	0	-2.027735	2.535544	2.687353
71	8	0	-2.674301	0.216623	0.341855
72	6	0	-2.615216	0.671401	-0.831115
73	6	0	-2.547311	2.094569	-1.085535
74	6	0	-1.944240	2.575699	-2.262087
75	7	0	-1.681567	1.676805	-3.293819
76	6	0	-2.023431	0.379621	-3.155287
77	6	0	-2.505535	-0.163903	-1.991938
78	6	0	-1.576722	3.924258	-2.354612
79	6	0	-1.774401	4.807311	-1.287531
80	6	0	-2.523621	4.318532	-0.179329
81	6	0	-2.886081	3.009092	-0.075927
82	6	0	-1.106259	2.143233	-4.542027
83	6	0	0.328133	2.585192	-4.545200
84	6	0	-0.064193	1.302296	-5.213044
85	6	0	-2.860822	-1.613961	-1.924150
86	8	0	-3.976436	-2.005310	-1.609394

87	9	0	-2.812351	5.131136	0.850225
88	7	0	-1.209407	6.066869	-1.337061
89	6	0	0.035570	6.248436	-2.083020
90	6	0	0.995185	7.157119	-1.306814
91	7	0	1.025842	6.814132	0.160252
92	6	0	-0.371379	6.985131	0.765286
93	6	0	-1.424880	7.098816	-0.334722
94	6	0	1.478868	5.359766	0.283525
95	6	0	1.828538	4.900906	1.685457
96	6	0	2.014208	7.703483	0.885953
97	6	0	1.817270	9.209015	0.667473
98	6	0	2.775467	9.840287	-0.352364
99	6	0	4.220410	9.882025	0.142161
100	8	0	0.049972	2.652046	0.369202
101	7	0	-1.876209	-2.455200	-2.382049
102	8	0	-2.206011	-3.802363	-2.404827
103	6	0	-2.873321	-4.127389	-3.624332
104	6	0	-0.437627	-2.284936	-2.193724
105	6	0	0.342079	-2.618874	-3.455913
106	7	0	1.935931	-0.625297	5.540037
107	8	0	0.886554	-0.404850	6.411812
108	6	0	0.054111	-1.561651	6.521892
109	6	0	3.216206	-0.361747	6.202209
110	6	0	3.577906	-1.465714	7.183769
111	8	0	-1.182112	4.477530	3.156007
112	1	0	-1.107233	4.286388	-3.258103
113	1	0	-3.359548	2.672617	0.838024
114	1	0	-1.862928	-0.235007	-4.035158
115	1	0	-0.135326	6.704250	-3.066207
116	1	0	0.503673	5.271720	-2.241131
117	1	0	-1.375650	8.072488	-0.831594
118	1	0	-2.415131	7.022690	0.101209
119	1	0	0.714154	8.205498	-1.395467
120	1	0	2.011534	7.044557	-1.690842

121	1	0	-0.565562	6.110285	1.390972
122	1	0	-0.363596	7.872682	1.399405
123	1	0	2.338621	5.259696	-0.385588
124	1	0	0.666972	4.748148	-0.107612
125	1	0	1.989227	3.820871	1.625003
126	1	0	2.742794	5.360877	2.065758
127	1	0	1.014361	5.045585	2.401243
128	1	0	1.908634	7.437748	1.938403
129	1	0	3.006704	7.379678	0.556124
130	1	0	1.972550	9.701376	1.633172
131	1	0	0.776174	9.425761	0.399182
132	1	0	2.728443	9.309736	-1.310737
133	1	0	2.434345	10.860714	-0.552423
134	1	0	4.869849	10.351112	-0.600519
135	1	0	4.295615	10.459009	1.068916
136	1	0	4.621802	8.882211	0.338978
137	1	0	-1.816172	2.679360	-5.165262
138	1	0	0.864256	2.498582	-3.604636
139	1	0	0.584888	3.439414	-5.162166
140	1	0	-0.092351	1.251441	-6.294753
141	1	0	0.248204	0.388300	-4.718446
142	1	0	-3.014807	-5.209040	-3.598304
143	1	0	-3.841005	-3.620324	-3.673407
144	1	0	-2.256100	-3.854083	-4.484383
145	1	0	-0.147267	-2.967316	-1.385796
146	1	0	-0.249094	-1.264842	-1.850005
147	1	0	1.414066	-2.485671	-3.285273
148	1	0	0.170846	-3.656463	-3.754739
149	1	0	0.046449	-1.969311	-4.285316
150	1	0	6.413364	-2.562278	0.700724
151	1	0	3.025704	0.863627	-0.512257
152	1	0	3.592355	-2.388646	4.354180
153	1	0	7.196371	0.499417	-2.088783
154	1	0	6.693525	-0.344093	-3.543555

155	1	0	8.300977	-2.488064	-0.768145
156	1	0	6.928397	-3.511488	-1.197054
157	1	0	9.275637	-0.746039	-1.929312
158	1	0	9.155761	0.058346	-3.501523
159	1	0	7.372888	-3.442393	-3.526852
160	1	0	8.833913	-3.984898	-2.679927
161	1	0	8.991278	-1.048838	-5.417621
162	1	0	7.421527	-1.724694	-4.952994
163	1	0	8.222247	-3.019700	-6.802529
164	1	0	9.816502	-3.274250	-6.105583
165	1	0	8.390884	-4.078227	-5.407421
166	1	0	10.532262	-3.327014	-4.072567
167	1	0	10.914688	-1.607093	-4.205892
168	1	0	11.760520	-3.296311	-2.159734
169	1	0	10.367896	-2.581963	-1.402208
170	1	0	11.395181	-0.259024	-1.980626
171	1	0	12.249074	-1.230050	-0.796756
172	1	0	13.834452	-0.229412	-2.437062
173	1	0	13.866536	-1.994764	-2.542665
174	1	0	13.026127	-1.042877	-3.773302
175	1	0	5.159429	-4.152108	2.339122
176	1	0	7.247877	-1.981687	3.120803
177	1	0	7.614166	-3.743145	2.764999
178	1	0	6.055270	-4.403648	4.669137
179	1	0	5.763801	-2.627746	4.991556
180	1	0	-0.668549	-1.308826	7.297349
181	1	0	-0.463759	-1.753461	5.578960
182	1	0	0.644260	-2.430756	6.827616
183	1	0	3.098786	0.593286	6.723779
184	1	0	3.976008	-0.226090	5.431929
185	1	0	4.514558	-1.222142	7.690812
186	1	0	2.798552	-1.567078	7.942981
187	1	0	3.695727	-2.432160	6.682203
188	1	0	-6.033925	-4.341130	0.062631

189	1	0	-4.389702	-0.545846	2.777277
190	1	0	-1.464944	-4.769696	-0.054886
191	1	0	-8.587702	-3.975186	-0.885864
192	1	0	-7.048437	-3.238431	-1.352571
193	1	0	-9.830670	-1.954530	0.598379
194	1	0	-8.808161	-1.211322	1.807383
195	1	0	-9.861778	-2.085975	-1.625356
196	1	0	-8.607448	-2.271480	-2.857037
197	1	0	-7.870638	0.374140	0.251246
198	1	0	-9.600283	0.348296	-0.129152
199	1	0	-6.685077	-1.030106	-2.780798
200	1	0	-6.342679	-1.153843	-1.055498
201	1	0	-5.169979	0.711554	-1.943697
202	1	0	-6.603408	1.468273	-2.637806
203	1	0	-6.396977	1.381300	-0.871517
204	1	0	-8.774411	1.319325	-2.159771
205	1	0	-8.582772	0.167132	-3.489169
206	1	0	-10.975437	1.185399	-2.725866
207	1	0	-10.995779	-0.191187	-1.662235
208	1	0	-10.655703	-0.268844	-4.707820
209	1	0	-10.698109	-1.706539	-3.703379
210	1	0	-12.937646	-1.283736	-4.697818
211	1	0	-13.025417	-1.118566	-2.939640
212	1	0	-12.985029	0.324476	-3.962999
213	1	0	-4.226141	-4.946625	-1.431192
214	1	0	-2.412097	-6.850878	0.251583
215	1	0	-3.092934	-7.202019	-1.406628
216	1	0	-5.438732	-6.939821	-0.441655
217	1	0	-4.693628	-6.561613	1.188499
218	1	0	1.312028	-5.184915	2.602862
219	1	0	-0.381805	-5.228652	2.115790
220	1	0	0.885189	-7.200130	1.188870
221	1	0	0.327706	-6.173993	-0.147001
222	1	0	2.017174	-6.104953	0.388649

223	1	0	3.481897	-3.150751	-0.439671
224	1	0	2.016150	-4.010815	-0.994551
225	1	0	1.962003	-2.235790	-0.673155

$(NO_2)_2 \cdot Lu(H_2O)_5^+$

Center Number	Atomic Number	Atomic Type	Coordinates (Angstroms) X	Y	Z
1	8	0	1.229930	2.201497	0.123586
2	71	0	-0.007089	0.173722	0.055354
3	8	0	-1.761872	-0.590155	-1.232435
4	7	0	-2.573388	-0.796646	-0.266754
5	8	0	-3.666029	-1.250294	-0.421144
6	8	0	-2.110861	-0.475845	0.886177
7	8	0	0.115901	0.439651	2.336984
8	8	0	1.670534	-0.837994	-1.174302
9	7	0	2.560935	-0.823118	-0.245546
10	8	0	3.645075	-1.298218	-0.389967
11	8	0	2.181155	-0.260341	0.832421
12	8	0	0.127044	1.181165	-2.090381
13	8	0	0.021693	-2.113287	0.582806
14	8	0	-1.388425	2.046445	0.197698
15	1	0	2.061526	2.187866	0.626272
16	1	0	-0.740719	1.318856	-2.508120
17	1	0	-0.726417	-2.522046	1.047010
18	1	0	-0.966721	2.878729	0.469180
19	1	0	0.892770	0.041100	2.766270
20	1	0	0.382376	-2.764231	-0.040954
21	1	0	0.654764	0.640305	-2.704114
22	1	0	-2.261604	1.993413	0.618738
23	1	0	1.425643	2.553045	-0.761961
24	1	0	-0.644310	0.336091	2.932112

$(NO_2)_2 \cdot Lu(4)_3^{+4}$

Center Number	Atomic Number	Atomic Type	Coordinates (Angstroms) X	Y	Z
1	6	0	-9.167996	-1.037647	-1.561554
2	7	0	-7.920071	-1.759231	-1.723349
3	6	0	-8.043094	-3.196362	-2.014774
4	6	0	-9.499728	-3.624027	-1.952424
5	7	0	-10.373602	-2.839564	-2.900881
6	6	0	-10.100924	-1.347130	-2.723179
7	6	0	-6.804947	-1.312076	-1.024640
8	6	0	-5.525974	-1.838085	-1.344260
9	6	0	-4.388507	-1.431143	-0.712653
10	6	0	-4.460538	-0.438478	0.280592
11	6	0	-5.710824	0.112804	0.610253
12	6	0	-6.874608	-0.329451	-0.041257
13	7	0	-5.775245	1.103216	1.592753
14	6	0	-4.647793	1.541420	2.180821
15	6	0	-3.399588	1.040502	1.912303
16	6	0	-3.237400	0.020094	0.913150
17	8	0	-2.132132	-0.475387	0.603435
18	71	0	-0.033426	0.398787	0.442914
19	8	0	1.572521	1.784304	1.805754
20	7	0	1.396725	2.798399	1.085088
21	8	0	1.797212	3.908162	1.390575
22	6	0	-7.041868	1.733454	1.921691
23	6	0	-8.076791	0.931122	2.655290
24	6	0	-7.343148	2.032202	3.358552
25	6	0	-2.177632	1.603568	2.531423
26	8	0	-1.171828	1.822140	1.835852
27	9	0	-5.449809	-2.747134	-2.326485
28	6	0	-10.031908	-3.251767	-4.327423
29	6	0	-10.921972	-2.638596	-5.395566

30	6	0	-11.835576	-3.158499	-2.638898
31	6	0	-12.308751	-2.903877	-1.201595
32	6	0	-12.474107	-4.175151	-0.356758
33	6	0	-13.652317	-5.033035	-0.814666
34	8	0	0.088157	-1.850179	0.102211
35	6	0	0.931789	-2.586993	0.659110
36	6	0	1.841497	-3.396106	-0.122629
37	6	0	2.800342	-4.219640	0.482435
38	7	0	2.845853	-4.296749	1.873283
39	6	0	2.070481	-3.491883	2.613480
40	6	0	1.110842	-2.656643	2.088294
41	6	0	1.876476	-3.226835	-1.514936
42	6	0	2.862735	-3.814672	-2.251592
43	6	0	3.873665	-4.626612	-1.663234
44	6	0	3.758923	-4.876909	-0.295674
45	7	0	4.986930	-5.097645	-2.345908
46	6	0	6.257600	-5.200261	-1.625618
47	6	0	7.381370	-4.491744	-2.395807
48	7	0	6.895713	-3.210463	-3.021303
49	6	0	5.812017	-3.538273	-4.047051
50	6	0	5.189719	-4.910272	-3.772424
51	6	0	6.353248	-2.314884	-1.906324
52	6	0	6.007344	-0.894883	-2.314113
53	6	0	8.039629	-2.484560	-3.707129
54	6	0	8.890761	-3.328995	-4.661413
55	6	0	10.184935	-3.883661	-4.056963
56	6	0	11.007336	-4.636272	-5.099144
57	9	0	2.907255	-3.536910	-3.571505
58	6	0	0.388285	-1.678515	2.929127
59	8	0	0.170321	-0.528228	2.508608
60	6	0	3.917470	-5.038047	2.506545
61	6	0	3.636511	-5.759951	3.784783
62	6	0	3.847458	-6.535465	2.516311
63	7	0	0.066805	-1.992681	4.201698

64	8	0	-0.543521	-0.991556	4.936703
65	6	0	0.418603	-0.051661	5.427898
66	6	0	-0.193444	-3.310564	4.781610
67	6	0	0.626908	-3.545645	6.041506
68	8	0	-1.517838	1.695292	-1.006459
69	7	0	-0.821061	1.413357	-2.014244
70	8	0	-0.038643	0.409646	-1.870677
71	8	0	2.196032	-0.281640	-0.096773
72	6	0	3.223655	0.438223	-0.026021
73	6	0	3.396849	1.602294	-0.865747
74	6	0	4.173991	2.682177	-0.408463
75	7	0	4.988445	2.496833	0.704468
76	6	0	5.068709	1.277541	1.281838
77	6	0	4.263770	0.223450	0.939130
78	6	0	4.066729	3.927988	-1.041840
79	6	0	3.194793	4.119984	-2.115185
80	6	0	2.574696	2.960944	-2.662123
81	6	0	2.641590	1.751186	-2.041221
82	6	0	5.780821	3.596649	1.220963
83	6	0	5.056331	4.732177	1.883784
84	6	0	5.870903	3.779469	2.705087
85	6	0	4.442119	-1.115268	1.584297
86	8	0	4.775720	-2.104185	0.944328
87	9	0	1.807485	3.066132	-3.761767
88	7	0	2.928751	5.410065	-2.543120
89	6	0	2.868441	6.465283	-1.530197
90	6	0	1.628135	7.341709	-1.738839
91	7	0	0.411057	6.523082	-2.084271
92	6	0	0.652363	5.758116	-3.386330
93	6	0	2.142660	5.718430	-3.726472
94	6	0	0.156935	5.544543	-0.934949
95	6	0	-1.178593	4.827887	-0.976969
96	6	0	-0.797054	7.426373	-2.238268
97	6	0	-0.610659	8.610958	-3.195023

98	6	0	-0.315222	9.953379	-2.511188
99	6	0	-1.509085	10.491606	-1.724337
100	8	0	0.766426	2.605105	0.003415
101	7	0	4.345525	-1.103642	2.950210
102	8	0	4.574052	-2.320768	3.575524
103	6	0	5.971291	-2.506384	3.800929
104	6	0	3.438632	-0.275073	3.741470
105	6	0	4.129164	0.319459	4.958606
106	7	0	-2.201307	1.947986	3.832101
107	8	0	-1.021560	2.452268	4.343974
108	6	0	-0.830141	3.823637	3.985315
109	6	0	-3.046324	1.407193	4.898845
110	6	0	-3.661617	2.511629	5.743349
111	8	0	-0.850924	2.051910	-3.041323
112	1	0	4.636138	4.769389	-0.671999
113	1	0	2.030997	0.934244	-2.410331
114	1	0	5.803383	1.190657	2.075903
115	1	0	3.747942	7.119645	-1.563719
116	1	0	2.829659	6.003496	-0.538787
117	1	0	2.481238	6.684186	-4.114098
118	1	0	2.316413	4.988849	-4.511571
119	1	0	1.778427	8.059561	-2.544021
120	1	0	1.401346	7.897137	-0.825981
121	1	0	0.263153	4.748503	-3.242729
122	1	0	0.081450	6.242987	-4.179284
123	1	0	0.253177	6.136579	-0.019957
124	1	0	0.966719	4.813443	-0.952297
125	1	0	-1.166024	4.103473	-0.159035
126	1	0	-2.026353	5.499256	-0.828415
127	1	0	-1.325245	4.239988	-1.887985
128	1	0	-1.596809	6.767652	-2.578029
129	1	0	-1.047918	7.766683	-1.228431
130	1	0	-1.540761	8.715537	-3.763528
131	1	0	0.158390	8.385102	-3.943541

132	1	0	0.555782	9.871737	-1.850600
133	1	0	-0.041456	10.675053	-3.287021
134	1	0	-1.269235	11.455735	-1.270151
135	1	0	-2.373221	10.633254	-2.380574
136	1	0	-1.811937	9.818602	-0.915210
137	1	0	6.656925	3.818067	0.618371
138	1	0	3.976779	4.636059	1.956871
139	1	0	5.435581	5.734037	1.714919
140	1	0	6.824218	4.104143	3.104217
141	1	0	5.318848	3.091923	3.337500
142	1	0	6.053508	-3.443909	4.353063
143	1	0	6.502944	-2.581619	2.848144
144	1	0	6.376144	-1.685572	4.399120
145	1	0	2.618543	-0.930236	4.059004
146	1	0	3.016104	0.494952	3.091416
147	1	0	3.423414	0.924925	5.533758
148	1	0	4.513432	-0.468956	5.611568
149	1	0	4.964962	0.960256	4.661674
150	1	0	-7.829880	0.086871	0.244895
151	1	0	-3.423881	-1.843280	-0.987743
152	1	0	-4.788196	2.350763	2.889950
153	1	0	-7.513302	-3.787505	-1.260566
154	1	0	-7.593480	-3.440595	-2.979516
155	1	0	-9.652915	-1.221431	-0.590149
156	1	0	-8.962566	0.034673	-1.622080
157	1	0	-9.895697	-3.473213	-0.948850
158	1	0	-9.604440	-4.680344	-2.209643
159	1	0	-9.634613	-0.995094	-3.643699
160	1	0	-11.057274	-0.838327	-2.595901
161	1	0	-10.091801	-4.342591	-4.349801
162	1	0	-8.992026	-2.959977	-4.481323
163	1	0	-10.468833	-2.859367	-6.364567
164	1	0	-11.925843	-3.064815	-5.400976
165	1	0	-10.995397	-1.550904	-5.312562

166	1	0	-12.391764	-2.548078	-3.349737
167	1	0	-11.964540	-4.208538	-2.921433
168	1	0	-13.279323	-2.401057	-1.259830
169	1	0	-11.646119	-2.188912	-0.700229
170	1	0	-11.555094	-4.773799	-0.373056
171	1	0	-12.627573	-3.876604	0.684540
172	1	0	-13.744918	-5.925645	-0.192062
173	1	0	-14.589389	-4.472684	-0.743838
174	1	0	-13.542885	-5.370241	-1.851057
175	1	0	-7.371973	2.442283	1.167784
176	1	0	-7.811152	-0.085955	2.924391
177	1	0	-9.115422	1.083931	2.384731
178	1	0	-7.861326	2.959446	3.570948
179	1	0	-6.617515	1.732758	4.108387
180	1	0	0.070213	4.125433	4.519958
181	1	0	-0.669341	3.921464	2.909661
182	1	0	-1.683700	4.425801	4.309679
183	1	0	-2.404129	0.757320	5.501482
184	1	0	-3.801189	0.769758	4.438896
185	1	0	-4.252307	2.076599	6.552878
186	1	0	-2.879496	3.130406	6.190177
187	1	0	-4.312839	3.158581	5.146372
188	1	0	4.463435	-5.542757	0.181002
189	1	0	1.161148	-2.564187	-1.991127
190	1	0	2.283074	-3.515388	3.676639
191	1	0	6.556761	-6.243653	-1.473811
192	1	0	6.152614	-4.735619	-0.643161
193	1	0	5.844787	-5.712342	-4.124597
194	1	0	4.257312	-5.004556	-4.320763
195	1	0	7.775327	-5.112839	-3.199722
196	1	0	8.204332	-4.241518	-1.722653
197	1	0	5.057985	-2.755175	-3.977499
198	1	0	6.262541	-3.502043	-5.039660
199	1	0	7.127006	-2.311270	-1.133481

200	1	0	5.470863	-2.799722	-1.490139
201	1	0	5.565825	-0.430460	-1.427675
202	1	0	6.878043	-0.303457	-2.600690
203	1	0	5.261100	-0.844904	-3.111831
204	1	0	7.565493	-1.656464	-4.236175
205	1	0	8.657460	-2.066739	-2.905424
206	1	0	9.159571	-2.679177	-5.500919
207	1	0	8.302546	-4.141489	-5.108197
208	1	0	10.769874	-3.048408	-3.654561
209	1	0	9.973595	-4.547383	-3.211163
210	1	0	11.937103	-5.011374	-4.665915
211	1	0	10.451983	-5.491549	-5.496891
212	1	0	11.264801	-3.983107	-5.938495
213	1	0	4.878042	-4.548382	2.371511
214	1	0	2.625731	-5.733072	4.180036
215	1	0	4.416771	-5.761386	4.536883
216	1	0	4.773713	-7.083787	2.387174
217	1	0	2.972176	-6.992549	2.066846
218	1	0	-1.263605	-3.339873	5.007930
219	1	0	0.000862	-4.058692	4.012160
220	1	0	0.397938	-4.529285	6.458194
221	1	0	1.702785	-3.495513	5.840475
222	1	0	0.384604	-2.794790	6.797765
223	1	0	-0.136843	0.596136	6.106113
224	1	0	1.211220	-0.573742	5.974177
225	1	0	0.820208	0.547337	4.605905

5. References

1. Gaussian 16, Revision B.01, M. J. Frisch; G. W. Trucks; H. B. Schlegel; G. E. Scuseria; M. A. Robb; J. R. Cheeseman; G. Scalmani; V. Barone; G. A. Petersson; H. Nakatsuji; X. Li; M. Caricato; A. V. Marenich; J. Bloino; B. G. Janesko; R. Gomperts; B. Mennucci; H. P. Hratchian; J. V. Ortiz; A. F. Izmaylov; J. L. Sonnenberg; D. Williams-Young; F. Ding; F. Lipparini; F. Egidi; J. Goings; B. Peng; A. Petrone; T. Henderson; D. Ranasinghe; V. G. Zakrzewski; J. Gao; N. Rega; G. Zheng; W. Liang; M. Hada; M. Ehara; K. Toyota; R. Fukuda; J. Hasegawa; M. Ishida; T. Nakajima; Y. Honda; O. Kitao; H. Nakai; T. Vreven; K. Throssell; J. A. Montgomery, Jr.; J. E. Peralta; F. Ogliaro; M. J. Bearpark; J. J. Heyd; E. N. Brothers; K. N. Kudin; V. N. Staroverov; T. A. Keith; R. Kobayashi; J. Normand; K. Raghavachari; A. P. Rendell; J. C. Burant; S. S. Iyengar; J. Tomasi; M. Cossi; J. M. Millam; M. Klene; C. Adamo; R. Cammi; J. W. Ochterski; R. L. Martin; K. Morokuma; O. Farkas; J. B. Foresman; D. J. Fox. Gaussian, Inc., Wallingford CT, 2016.
2. Density-Functional Theory of Atoms and Molecules, R. G. Parr; W. Yang. 1989, Oxford, New York.
3. Y. Zhao, D. G. Trohlar. The M06 suite of density functionals for main group thermochemistry, thermochemical kinetics, noncovalent interactions, excited states, and transition elements: two new functionals and systematic testing of four M06-class functionals and 12 other functionals. *Theor. Chem. Acc.* **2008**, *120*, 215–241.
4. Modern Theoretical Chemistry Vol. 3, Dunning, T. H. Jr.; Hay, P. J. in , Ed. Schaefer III, H. F. 1977. Plenum Press, New York.
5. S. Grimme, J. Antony, S. Ehrlich, H. J. Krieg. A consistent and accurate *ab initio* parametrization of density functional dispersion correction (DFT-D) for the 94 elements H-Pu *Chem. Phys*. **2010**, *132*, 154104.
6. R. Cammi, B. Mennucci, J. Tomasi. Fast Evaluation of Geometries and Properties of Excited Molecules in Solution: A Tamm-Dancoff Model with Application to 4-Dimethylaminobenzonitrile. *J. Phys. Chem. A* **2000**, *104*, 5631-5637.
7. J. Tomasi, B. Mennucci, R. Cammi. Quantum Mechanical Continuum Solvation Models*Chem. Rev.* **2005**, *105*, 2999-3093.
